# Trogocytosis of CAR molecule regulates CAR-T cell dysfunction and tumor antigen escape

**DOI:** 10.1038/s41392-023-01708-w

**Published:** 2023-12-25

**Authors:** You Zhai, Yicong Du, Guanzhang Li, Mingchen Yu, Huimin Hu, Changqing Pan, Di Wang, Zhongfang Shi, Xu Yan, Xuesong Li, Tao Jiang, Wei Zhang

**Affiliations:** 1 Department of Molecular Neuropathology, Beijing Neurosurgical Institute, Capital Medical University, Beijing, PR China; 2 Department of Neurosurgery, Beijing Tiantan Hospital, Capital Medical University, Beijing, PR China; 3 Department of Urology, Peking University First Hospital, Institute of Urology, Peking University, National Urological Cancer Center, Beijing, PR China; 4 Department of Pathophysiology, Beijing Neurosurgical Institute, Capital Medical University, Beijing, PR China; 5 China National Clinical Research Center for Neurological Diseases, Beijing, PR China; 6 Center of Brain Tumor, Beijing Institute for Brain Disorders, Beijing, PR China; 7 Research Unit of Accurate Diagnosis, Treatment, and Translational Medicine of Brain Tumors, Chinese Academy of Medical Sciences, Beijing, PR China; 8 Chinese Glioma Genome Atlas Network (CGGA) and Asian Glioma Genome Atlas Network (AGGA), Beijing, PR China

**Keywords:** Immunotherapy, Tumour immunology

## Abstract

Chimeric antigen receptor (CAR) T-cell therapy has demonstrated clinical response in treating both hematologic malignancies and solid tumors. Although instances of rapid tumor remissions have been observed in animal models and clinical trials, tumor relapses occur with multiple therapeutic resistance mechanisms. Furthermore, while the mechanisms underlying the long-term therapeutic resistance are well-known, short-term adaptation remains less understood. However, more views shed light on short-term adaptation and hold that it provides an opportunity window for long-term resistance. In this study, we explore a previously unreported mechanism in which tumor cells employ trogocytosis to acquire CAR molecules from CAR-T cells, a reversal of previously documented processes. This mechanism results in the depletion of CAR molecules and subsequent CAR-T cell dysfunction, also leading to short-term antigen loss and antigen masking. Such type of intercellular communication is independent of CAR downstream signaling, CAR-T cell condition, target antigen, and tumor cell type. However, it is mainly dependent on antigen density and CAR sensitivity, and is associated with tumor cell cholesterol metabolism. Partial mitigation of this trogocytosis-induced CAR molecule transfer can be achieved by adaptively administering CAR-T cells with antigen density-individualized CAR sensitivities. Together, our study reveals a dynamic process of CAR molecule transfer and refining the framework of clinical CAR-T therapy for solid tumors.

## Introduction

The adoptive transfer of engineered T cells is a form of immunotherapy that has been demonstrated to induce clinical response in patients with various blood and solid malignancies.^1–5^ Nevertheless, consistently impressive clinical results in glioblastoma multiforme (GBM) and other solid tumors have yet to be reported, with most CAR-T-treated patients experiencing progression or relapse shortly after treatment.^1,6–8^ Among the adequate reports of the relapses in liquid malignancies post CAR-T treatment, CD19 positive relapses are related with CAR-T expansion or persistence, while CD19 negative relapses (20–30% of patients) are related with the adaptation of tumor cells under the pressure of functioning CAR-T cells.^9^ Meanwhile, CD19 negative relapses produced the worst prognosis with limited subsequent treatments.^10^ Although lack of evidence in solid tumors corroborate such view of liquid tumors, these clinical studies indicate that apart from developing novel CAR-T cells to reduce relapse rates,^11–13^ a deeper understanding of the tumor cell inherent adaptation mechanisms^14^ is needed. Therefore, despite the importance of developing optimal CAR-T cells based on the deeper understanding of T cell characteristics, a comprehensive understanding of the target cells and the mechanisms of the inherent and acquired resistance to CAR-T therapy is necessary.^9,15^

Tumor cell characteristic is one of the important determinants of CAR-T therapy clinical outcome and CAR-T resistance.^16^ This issue includes antigen-dependent and antigen-independent mechanisms.^17^ Antigen-dependent resistance is a more common and key mechanism of relapse in GBM and other solid tumors subjected to CAR-T therapy, characterized as the emergence of tumors with reduced, modified, or impaired expression of the target antigen.^18,19^ Some of the mechanisms of this issue, such as expression downregulation, lineage switching,^20^ and splice variants,^21^ have been widely explored. Meanwhile, the comprehensive tumor-intrinsic anti-apoptosis modification, which contributes to antigen-independent treatment failure,^22,23^ has also been reported. Despite these long-term, late-start mechanisms, recent studies shed light on another therapeutic resistance, characterized by the transient and short-term antigen escape, such as protein transfer^24,25^ and antigen depletion.^26^ Although the underlying mechanisms remain less understood, these phenomenological and functional researches indicate that these phenomena may attenuate CAR-T and other immune cell activity and provide an opportunity window for such transcriptomic or genome adaptation to occur.

Trogocytosis,^27^ is an intercellular material transfer mechanism in a cell-contact-dependent manner. Acceptor cells acquire and internalize the membrane of donor cells, along with their associated molecules. Such phenomenon was originally observed at the immunological synapse between T cell and antigen presenting cells (APC),^28^ where T cell internalized antigen-MHC complexes through invaginating the plasma membrane of APC. And, the contact site is of higher accumulation of TCRs and MHC.^29^ This issue has been well documented in both myeloid and lymphoid lineages in recent years. Although whether trogocytosis and phagocytosis are the same is still under debate, more researches unveiled that the acceptor cells may temporarily perform functions similar to those of the donor cells^30,31^ and may be regulated by the intracellular signaling triggered by trogocytosed molecules.^32^ These results suggest that such ligand-receptor triggered intercellular gnawing may differ from the concept of phagocytosis.^33,34^ Meanwhile, recent studies have broadened the field of trogocytosis. In addition to classical ligand-receptor-induced trogocytosis, such as CD80-CD28,^35^ and MHC-TCR,^36^ antibodies and CAR molecules have also been found to mediate the same effects.^24,37–39^ These novel findings reveal the comprehensive modulation of trogocytosis, that it can induce fratricide effect of trogocytotic T cells,^40^ as well as CAR-T cells,^24^ or reduce antigen density of tumor cells,^41^ all of these benefit tumor immune escape and was considered to be one of the key mechanisms in the failure of CAR-T cell therapy.^42^

In most related studies, T, CAR-T and other lymphoid cells have been reported as acceptors, and are considered to promote tumor immune escape via trogocytosis. Here, we present the novel finding that CAR-T cells can also serve as donors, transfer CAR molecules to tumor cells. Such phenomenon results in the depletion of CAR molecule in CAR-T cell and the down-regulation of downstream signaling. It also dampens serial killing, and contributes to tumor cell antigen-masking. We further demonstrate that the extent of CAR molecule transfer depends on antigen density and CAR sensitivity, rather than target antigen, CAR molecule signaling, or immune checkpoint expression. Such CAR molecule transfer can be counterbalanced by adjusting CAR sensitivity based on antigen density, which high-affinity CAR molecule for low-antigen density tumor cells, and low-affinity CAR molecule for high-antigen density tumor cells. Meanwhile, we preliminarily explored the underlying mechanism of CAR molecule trogocytosis and initially considered that such phenomenon is positively correlated with tumor cell cholesterol production. Altogether, our study not only unveils the existence of the reverse process of the previous reported trogocytosis, but also provides an antigen-dependent CAR-T administration strategy to counterbalance the corresponding immune escape. Unlike previous straightforward thought that higher affinity CAR molecule would perform better, results from this study provide a more flexible strategy and broaden the understanding of CAR-T therapy.

## Results

### CAR-T cell membrane and CAR molecules are transferred to tumor cells

**Fig. 1:**
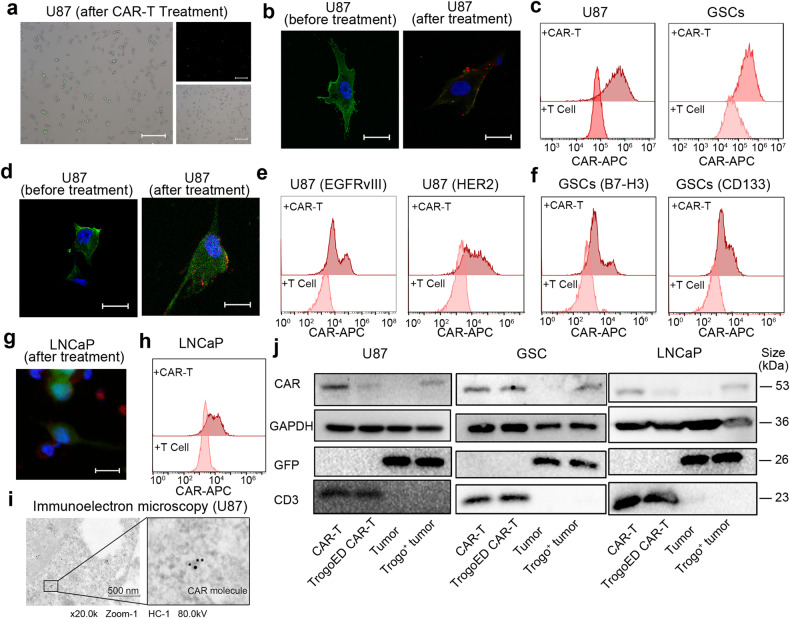
Transfer of CAR-T cell membrane and CAR molecules to target tumor cells. **a** Green fluorescent dye pre-labeled CAR-T cells were cocultured with GSCs overnight and subsequently removed. Representative images show partially stained GSCs (Scale bar: 100 μm). **b** Green fluorescent dye-labeled EGFRvIII^+^ U87 cells were cocultured with or without red fluorescent pre-labeled EGFRvIII-target CAR-T cells. Representative images display red dye remained in U87 cells after cocultured with CAR-T cells (Scale bar: 10 μm). **c** Green fluorescent dye-labeled U87 cells or GSCs were cocultured with corresponding deep red fluorescent dye-labeled CAR-T cells or T cells overnight. Representative flow cytometry indicates that more deep red dye remained on the tumor cell after CAR-T treatment, than T cell treatment. Gate on tumor cells. Data are from at least three independent experiments. **d** EGFRvIII-eGFP fusion protein overexpressed U87 cells were cocultured with or without EGFRvIII-target CAR-T cells. CAR molecule (FLAG-tag labeled) was stained with Alexa Fluor 594 labeled antibody. Representative images show CAR molecule (red) positive U87 cells (green) after CAR-T treatment (Scale bar: 5 μm). **e**, **f** U87 cells (EGFRvIII overexpressed) and GSCs were cocultured with T cells or CAR-T cells (EGFRvIII, HER2, B7-H3, CD133 targeted) overnight. CAR molecule (FLAG-tag labeled) was stained with an anti-FLAG-tag (APC) antibody. Gate on tumor cells. Representative flow cytometric results display CAR molecules in tumor cells. Data are from at least six independent experiments. **g** Luciferase-eGFP expressed LNCaP cells were cocultured with PSMA-targeted CAR-T cells. CAR molecule (FLAG-tag labeled) was stained with Alexa Fluor 594 labeled antibody. Representative images show CAR molecules (red) transferred to LNCaP cells (green) (Scale bar: 5 μm). **h** LNCaP cells were cocultured with T cells or PSMA-target CAR-T cells overnight. CAR molecule (FLAG-tag labeled) was stained with an anti-FLAG-tag (APC) antibody. Gate on LNCaP cells. Representative flow cytometric results display the transfer of CAR molecules. Data are from at least six independent experiments. **i** Electron microscopy micrographs of EGFRvIII-target CAR-T cell treated U87 cells (EGFRvIII overexpressed). CAR molecule (FLAG-tag labeled) was stained with colloidal gold-labeled anti-FLAG tag antibody. Data are represented among at least four independent experiments. (Scale bar: 500 nm) **j** CAR molecule acquiring of tumor cells and loss of CAR-T cells were determined after effector and target cell separation. Indicated CAR-T cells were cocultured with GFP-labeled tumor cells (U87, GSCs, LNCaP) overnight before separation. Images are represented among two to three independent duplications

CAR-T cells were labeled with green fluorochromes on their membrane and cocultured with glioma stem cells (GSCs) overnight, which was followed by the removal of the CAR-T cells. The remaining GSCs exhibited partial green fluorescence (Fig. 1a). Confocal microscopy, live imaging, and flow cytometry provided further support for the transfer of CAR-T cell membranes to the GSCs and U87 cells (Supplementary Movie 1, Fig. 1b, c).

Considering the rapid expression of the CAR molecule and CAR-antigen binding, we hypothesized that the CAR molecule was the most likely candidate among the membrane proteins for undergoing transfer. We constructed EGFRvIII-targeted CAR-T cells and observed that the surviving GBM cell line (U87) acquired the CAR molecule, under the pressure of CAR-T cells (Fig. 1d). Additional evidence was found in both U87 and GSCs following treatment with corresponding CAR-T cells targeting EGFRvIII, HER2, B7-H3, and CD133, respectively (Fig. 1e, f). In order to visualize the occurrence of CAR molecule trogocytosis, we utilized T cells expressing an EGFRvIII CAR-mScarlet fusion, which enabled microscopic tracking. We discovered that CAR molecules were transferred to U87 cells overexpressing EGFRvIII-GFP fusion via immune synapses (Supplementary Movie 2). We then investigated prostate-specific membrane antigen-positive (PSMA^+^) LNCaP prostate tumor cells and observed the anticipated transfer of CAR molecules (Supplementary Movie 3, Fig. 1g, h). To minimize the impact of cell aggregation, we employed cell pellet filtration and a meticulous gating strategy (Supplementary Fig. 1a–c). Further, Electron microscopy of EGFRvIII-overexpressing U87 cells following EGFRvIII-targeted CAR-T treatment revealed the presence of CAR molecules within the U87 cell membrane (Fig. 1i). However, we did not observe CAR molecule transfer in CD19 CAR-T cells against Daudi cells (Supplementary Fig. 1d). Consistent with previous reports, we again observed antigen transfer, as the transfer of EGFRvIII-GFP fusion protein from U87 cells to CAR-T cells was detected (Supplementary Fig. 2a).

To strengthen our findings of CAR molecule transfer, tumor cells (GFP overexpressed) and corresponding T cells were separated for immunoblotting assays after coculture. Both CAR molecule loss in CAR-T cells, and CAR molecule acquisition in tumor cells were detected (Fig. 1j). Such trend increased with prolonged coculture time (Supplementary Fig. 2b). Together with the results of immunoblotting, confocal microscopy images also suggest that CAR molecules are acquired by tumor cells, not CD3 (Supplementary Fig. 2c). Collectively, these findings suggest that the CAR-T cell membrane, particularly the CAR molecule, is transferred to target cells via immune synapses during CAR-T-tumor cell interactions, with the process being more prevalent in solid tumors.

### CAR molecule trogocytosis is correlated with the characteristics of the CAR molecule and the target antigen

**Fig. 2:**
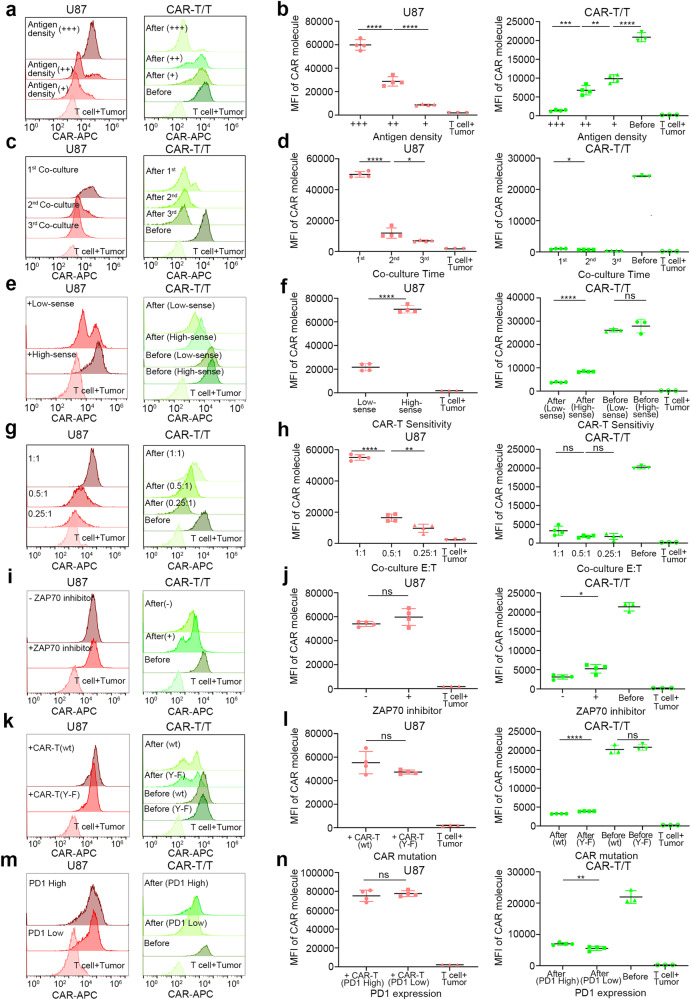
Trogocytic CAR molecule transfer is related to several factors. **a**, **b** U87 cells with different EGFRvIII expression levels were treated with EGFRvIII-targeted CAR-T cells or T cells. Gate on U87 cells (left) and CAR-T cells (right). Flow cytometry results indicate that both CAR molecule acquisition of tumor cells and the loss of CAR-T cells are antigen-density dependent. The mean fluorescence intensity (MFI) of CAR molecules (FLAG-tag labeled) of tumor cells and CAR-T/T cells are presented in **b**. **c**, **d** EGFRvIII-targeted CAR-T cells were successively cocultured with fresh U87 cells of EGFRvIII expression (high antigen density), for a total of three times. CAR molecule transfer was robust in the first stimulation and decreased progressively in subsequent stimulations. Meanwhile, rapid CAR molecule loss was observed in CAR-T cells. Additional replicates are displayed in **d**. **e**, **f** U87 cells of EGFRvIII expression (high antigen density) were cocultured with CAR-T cells of high sensitivity (High-sense, EGFRvIII scFv-CD28-CD3ζ) or low sensitivity (Low-sense, EGFRvIII scFv-CH2CH3-CD28-CD3ζ) overnight. Data reveal that CAR molecule trogocytosis depends on CAR molecule sensitivity. More replicates are shown in **f**. **g**, **h** U87 cells of EGFRvIII expression (high antigen density) were cocultured with CAR-T cells (high sensitivity) at indicated effector-to-target (E: T) ratios. Data reveal that CAR molecule acquisition of tumor cells is positively correlated with the E: T. CAR molecule loss in CAR-T cells was also observed. Independent replicates are presented in **h**. **i**, **j** U87 cells with a high density of EGFRvIII were cocultured with CAR-T cells (high sensitivity) supplemented with (+) or without (−) ZAP70 inhibitor. No significant biological difference was observed between (+) and (−) groups. The result indicates that CD3ζ downstream signaling is not relevant to CAR molecule transfer. Independent replicates are displayed in **j**. **k**, **l** Tyrosine residues of ITAMs were point-mutated to phenylalanine (Y–F) to silence downstream signaling. Mutate (Y-F) or wild type (wt) CAR-T cells (EGFRvIII-targeted, high sensitivity) were cocultured with U87 cells with a high density of EGFRvIII. Again, data demonstrate that trogocytosis is independent of CAR molecule downstream signal. Independent replicates are shown in **l**. **m**, **n** CAR-T cells (EGFRvIII-targeted, high sensitivity) were pre-induced to express PD1 before coculture with U87 cells (high density of EGFRvIII). Data indicate that trogocytic CAR molecule transfer is independent of PD1 expression. Additional replicates are provided in **n**. Data are represented as mean ± SD, unpaired Student’s *t* test, *P < 0.05; **P < 0.01; ***P < 0.001, ****P < 0.0001

We initially postulated that the transfer of CAR molecules was related to antigen density, based on previous research.^24^ We generated three U87 cell lines with varying EGFRvIII expression levels (Supplementary Fig. 2d). In addition, to maximize the extent of CAR molecule transfer, we constructed a CAR molecule with CD28 transmembrane domain and CD28 costimulatory domain^43,44^ (Supplementary Table 1). Flow cytometry analysis revealed that U87 cells expressing the highest levels of the target antigen acquired the greatest number of CAR molecules following CAR-T treatment (Fig. 2a, left), in comparison to the other two antigen expressing levels. Concurrently, CAR-T cells experienced rapid loss of CAR molecules after coculture (Fig. 2a, right). Multiple experiments demonstrated a positive correlation between antigen density and CAR molecule acquisition by U87 cells, as well as CAR molecule loss in CAR-T cells (Fig. 2b).

To explore other potential factors, we considered several variables. We repeatedly stimulated CAR-T cells with freshly cultured EGFRvIII^+^ U87 cells and examined the CAR molecules in both the target and effector cells following each coculture. Flow cytometry assays revealed the robust trogocytosis of CAR molecules from newly generated CAR-T cells by U87 cells. Concurrently, CAR-T cells rapidly lost CAR molecules upon initial tumor stimulation. In subsequent cocultures, residual CAR molecules decreased, and U87 cells acquired less of them (Fig. 2c, d).

We also assessed the impact of antigen sensitivity, a crucial factor for both trogocytosis^39^ and CAR-T efficiency.^44^ We constructed CAR-T cells with reduced antigen sensitivity by incorporating a CH2CH3 spacer domain.^43,45^ Interestingly, EGFRvIII^+^ U87 cells acquired more CAR molecules from CAR-T cells with higher antigen sensitivity compared to those with lower sensitivity (Fig. 2e, f). Replicate experiments showed similar trends in GSCs (Supplementary Fig. 3a, b) and LNCaP (Supplementary Fig. 4a, b) cells.

Furthermore, we examined the effect of the effector-to-target (E: T) cell ratio. We found that the acquisition of CAR molecules increased with a higher proportion of CAR-T cells (Fig. 2g, h), while robust CAR molecule loss persisted with a lower effector-to-target cell ratio. The same trend was observed in GSCs (Supplementary Fig. 3c, d) and LNCaP cells (Supplementary Fig. 4c, d).

We also investigated whether the activation of downstream signaling pathways in CAR molecules could regulate their transfer. We inhibited CD3ζ downstream signaling by adding ZAP180013 (a ZAP70 phosphorylation inhibitor) or by mutating tyrosine residues within ITAMs.^46^ No significant biological difference of CAR molecule transfer was observed between the inhibition conditions and negative control (Fig. 2i–l). The same trend was replicated in GSCs cocultured with low-sensitivity (Supplementary Fig. 3e, f) or high sensitivity (Supplementary Fig. 3g, h) B7-H3-targeted CAR-T cells, and LNCaP cells (Supplementary Fig. 4e, f).

Immune checkpoint expression has been considered a barrier to the efficacy of CAR-T therapy. We induced PD1 expression using conditioned medium (Supplementary Fig. 5a) and found that tumor cells acquired similar amounts of CAR molecules, regardless of PD1 expression (Fig. 2m, n). The same trend was observed in GSCs, independent of CAR sensitivity (Supplementary Fig. 3i, l), and LNCaP (Supplementary Fig. 4g, h). In addition, the mentioned cell dye fluorescence and CAR expression of CAR-T cells were also characterized (Supplementary Fig. 5b–d).

We then explored the effect of different transmembrane domains on trogocytosis. To minimize the synergistic effect of the transmembrane domain with co-stimulatory domain,^47,48^ and further compare the effect of different costimulatory domains (4-1BB and CD28), we unified the transmembrane domain as CD8a (Supplementary Fig. 5e). No significant biological difference in CAR molecule transfer was observed between the two sets of CAR-T cells (Supplementary Fig. 5f). Meanwhile, we preliminarily investigated the direction and the extent of the mutual molecule transfer between CAR-T and tumor cells. Fluorescent protein was used to detect CAR molecule or antigen (EGFRvIII) transfer (Supplementary Fig. 5g). Molecule trafficking was observed between the effector and target cells. CAR-T cells rapidly lost CAR molecule and acquired tumor antigen. Meanwhile, tumor cells underwent the opposite process to CAR-T cells (Supplementary Fig. 5h). These results indicate that there may be a dynamic balance in such intercellular interaction.

Finally, to mimic trogocytosis in vivo, we treated an animal model of high tumor burden with EGFRvIII-targeted (against U87 EGFRvIII high density) or B7-H3-targeted CAR-T (against GSCs) and harvested the tumor tissue two days post-infusion (Supplementary Fig. 6a). In accordance with our previous findings, tumor cells acquired more CAR molecules following high sensitivity CAR-T cell treatment (Supplementary Fig. 6b, c).

Collectively, these results suggest that CAR molecule transfer is primarily associated with the characteristics of the CAR-target interaction, including the relative abundance of each molecule and the sensitivity of the CAR molecule.

### Trogocytosis promotes the therapeutic resistance of tumor cells

**Fig. 3:**
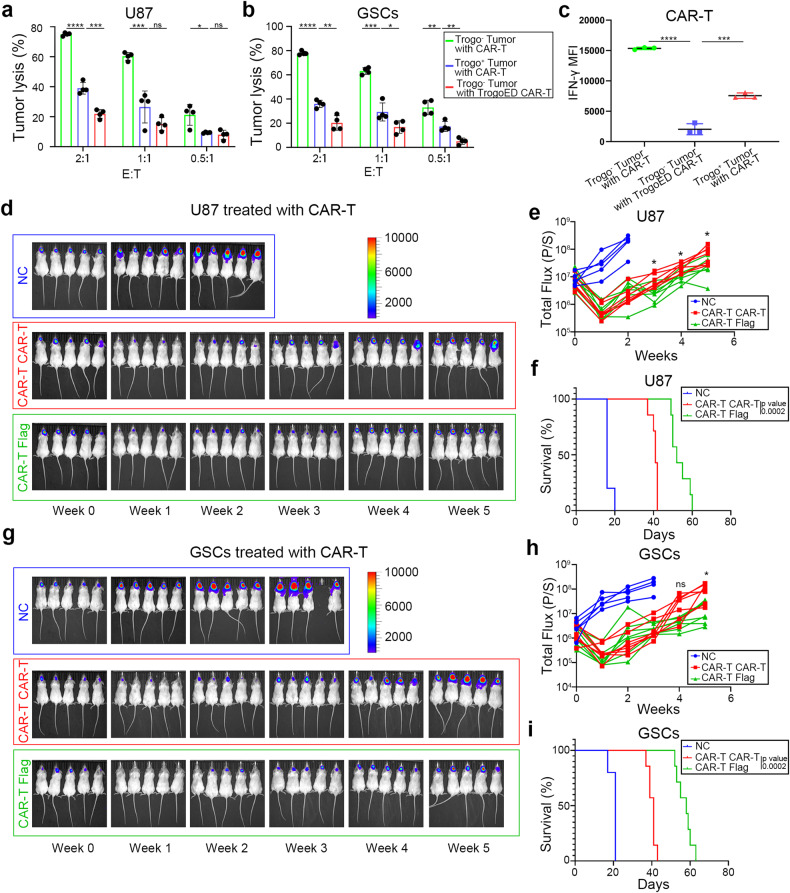
Trogocytosis of CAR molecule dampens CAR-T cytotoxicity. **a**, **b** U87 cells (high density of EGFRvIII) or GSCs were pre-treated with corresponding EGFRvIII-targeted or B7-H3 targeted CAR-T cells, respectively. Fresh tumor cells were cocultured with fresh CAR-T cells (Trogo^-^ Tumor with CAR-T, green column), as positive control. For the experimental group, tumor cells and CAR-T cells were pre-cocultured overnight. Survived tumor cells (Trogo^+^ Tumor) were isolated and further cocultured with fresh CAR-T cells (blue column); meanwhile, CAR-T cells were isolated and cocultured with fresh tumor cells (Trogo^-^ Tumor with TrogoED CAR-T, red column). The cytotoxicity assays demonstrate the reduced killing efficiency of TrogoED CAR-T and increased killing resistance of Trogo^+^ Tumor (*n* = 4 independent duplications). **c** Intracellular IFN-γ detection of U87-CAR-T coculture. Gate on CAR-T cells. Both TrogoED CAR-T cells (Trogo^-^ Tumor with TrogoED CAR-T, blue) and fresh CAR-T cocultured with trogo^+^ U87 cells (Trogo^+^ Tumor with CAR-T, red) expressed less IFN-γ (*n* = 3 independent duplications). **d** Representative bioluminescent image of tumor burden. U87 cells (high density of EGFRvIII) were intracranially injected into the frontal lobe following CAR-T treatment: NC, peripheral blood T cells; CAR-T CAR-T, two consecutive doses of EGFRvIII-targeted CAR-T; CAR-T Flag, one dose of EGFRvIII-targeted CAR-T followed by one dose of FLAG-tag targeted CAR-T. The second dose of CAR-T cells were injected two weeks after the first administration. **e** Bioluminescence intensity (total flux) of mice in **d**, *n* = 5–7 per group. **f** Kaplan–Meier analysis of CAR-T cell-treated mice. **g–i** The above in vivo experiments were replicated in B7-H3-targeted CAR-T cells against GSCs. Data are represented as mean ± SD, unpaired Student’s *t* test, *P < 0.05; **P < 0.01; ***P < 0.001, ****P < 0.0001

Our investigations revealed the occurrence and the existence of CAR molecule transfer. To explore the implications of such phenomenon in tumor immuno-therapeutic resistance, we isolated tumor cells and CAR-T cells post-coculture (Trogo^+^ Tumor, TrogoED CAR-T, respectively) for further cytotoxicity assays. The newly generated CAR-T cells failed to adequately activate antigen-dependent cytotoxicity and downstream signal, demonstrating reduced efficacy against Trogo^+^ Tumor compared to freshly cultured (Trogo^-^) tumor cells. Meanwhile, cytotoxicity and the downstream signal of activation against with Trogo^-^ tumor cells were also reduced in TrogoED CAR-T cells (Fig. 3a–c, Supplementary Fig. 6d). Furthermore, as expected, there were fewer antigen for the single-chain variable fragments (scFv, the same clone as the CAR molecule) binding, despite antigen expression of tumor cells remaining relatively stable post-coculture (Supplementary Fig. 6e). These results suggest that CAR molecule transfer may promote tumor resistance against CAR-T cell and hamper CAR-T cytotoxicity, as a consequence of decreasing recognizable antigen by the same antibody (scFv) in tumor cells (antigen-masking) and “depriving” CAR-T cells of CAR molecule (weakening downstream signal). Hence, we hypothesized that trogocytosis might contribute to tumor recurrence and short-term CAR-T therapeutic resistance, and that transferred CAR molecules could represent a novel antigen for salvaging CAR-T therapy.

We then generated anti-FLAG-tag CAR-T cells using the M2 antibody. To assess the impact of trogocytosis on CAR-T therapy relapse, we administered a conventional dose of high sensitivity CAR-T cells (approximately 10^6^ CAR-T cells) to immunocompromised mice with a substantial tumor burden (total flux ~10^7^). CAR-T cells were administered every other week for a total of 2 doses to minimize the effect of fratricide from the latter dose to the previous dose (Supplementary Fig. 6f). Animals received a single dose of either EGFRvIII-targeted (for U87, EGFRvIII high density) or B7-H3-targeted (for GSCs) CAR-T (high sensitivity) treatment, followed by an additional dose of either the previous CAR-T or FLAG-tag-targeted CAR-T cells for salvage therapy (CAR-T CAR-T group and CAR-T Flag group, respectively). As expected, the initial CAR-T infusion reduced tumor burden, though tumors recurred within two weeks post the first infusion. Intriguingly, a second dose of EGFRvIII-targeted or B7-H3-targeted CAR-T cells failed to control tumor growth, whereas FLAG-tag-targeted CAR-T cells delayed intracranial tumor progression and extended animal survival to a certain extent (Fig. 3d–i). Replicate experiments showed similar trends in subcutaneous LNCaP tumor models (Supplementary Fig. 6g). Collectively, these results provide evidence that CAR molecule trogocytosis may contribute to the therapeutic resistance of tumor cells to the consecutive doses of CAR-T therapy.

### Togocytosis of the CAR molecule is associated with cholesterol metabolism of tumor cells

We next to initially investigate the underlying mechanism of CAR molecule trogocytosis. We hypothesized that the state of the tumor cell membrane may contribute to CAR molecule transfer. Cholesterol, a well-known lipid for cell membrane stability and stiffness, has been highlighted in cancer immunology.^49^ Cholesterol depletion is a key protective factor for the cytotoxicity of T cells,^50,51^ and, to a certain extent, improves the prognosis of patients.^52–54^ We assumed that depleting the cholesterol of tumor cells may prevent CAR molecule trogocytosis.

**Fig. 4:**
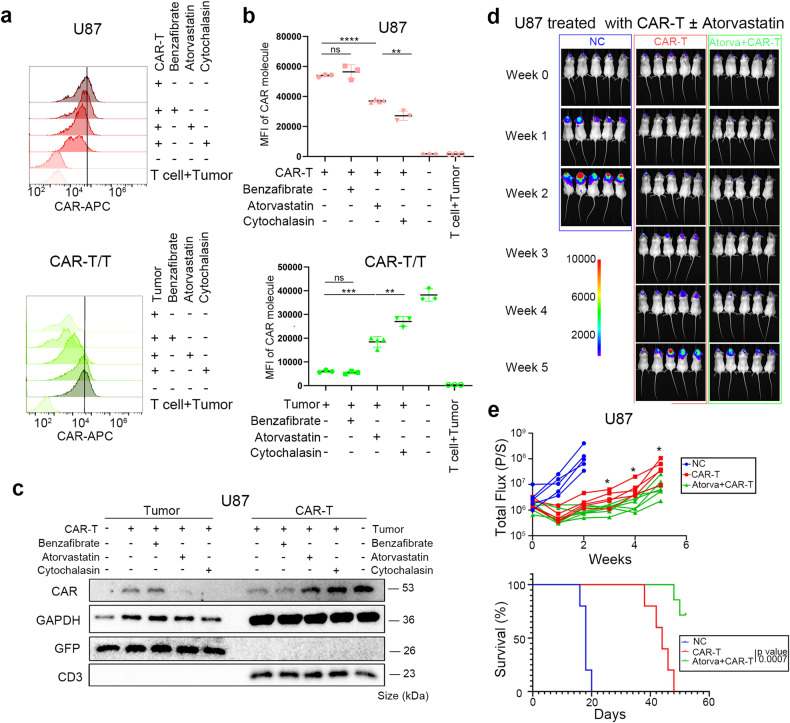
Trogocytosis is associated with cholesterol metabolism and can be partially offset by inhibiting cholesterol production. **a**, **b** U87 cells with high EGFRvIII density were treated with EGFRvIII-targeted CAR-T cells (high sensitivity) or T cells, supplemented with indicated drugs. Gate on U87 cells (up) and CAR-T cells (down). Flow cytometric data reveal that both atorvastatin and cytochalasin mitigated the CAR molecule acquisition of tumor cells and the CAR molecule loss of CAR-T cells. Additional replicates and statistical differences are provided in **b**. **c** U87 cells with high EGFRvIII density were cocultured with EGFRvIII-targeted CAR-T cells, supplemented with indicated drugs. Cells were magnetically separated for CAR molecule detection. Representative immunoblotting image from three independent duplications shows that atorvastatin and cytochalasin alleviate CAR molecule transfer. **d** Representative bioluminescent image of tumor burden. U87 cells (high density of EGFRvIII) were intracranially injected into the frontal lobe, following two doses of EGFRvIII-targeted CAR-T (high sensitivity) administration. Atorvastatin was orally supplemented. NC, peripheral blood T cells. **e**, **f** Bioluminescence intensity (total flux) and Kaplan–Meier analysis of mice in **d**, *n* = 5–7 per group. Data are represented as mean ± SD, unpaired Student’s *t* test, *P < 0.05; **P < 0.01; ***P < 0.001, ****P < 0.0001

To explore this, tumor cells were treated with atorvastatin prior to CAR-T treatment, while cytochalasin was used as the positive control.^25,55^ As expected, tumor cells acquired fewer CAR molecules, while CAR-T cells retained more CAR molecules post-coculture (Fig. 4a–c). The same trend was shown in GSCs (Supplementary Fig. 7a, b) and LNCaP cells (Supplementary Fig. 7c, d). Further, consistent with previous reports,^51,56,57^ atorvastatin produced a better outcome in GBM (Fig. 4d, e) and prostate tumor (Supplementary Fig. 7e) animal models. These results suggest that atorvastatin may enhance the antitumor response via inhibiting CAR molecule transfer and subsequently promoting CAR-T cell cytotoxicity.

### Tuning the sensitivity of CAR molecules to match tumor antigen density might benefit CAR-T therapy

**Fig. 5:**
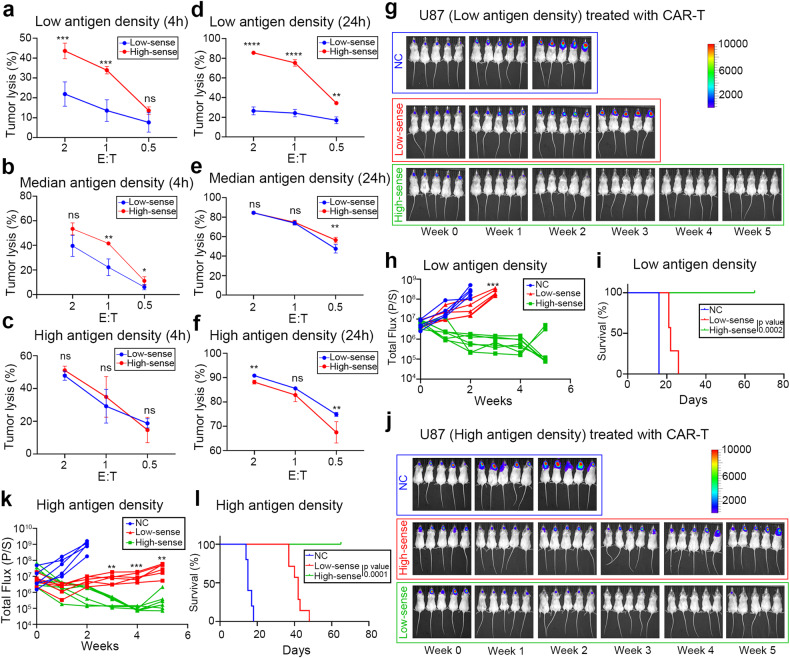
Trogocytosis-associated CAR-T resistance can be partially offset by adoptively administering CAR-T of antigen-density-matched sensitivity. **a**–**c** Short-term killing (4 h) of high or low-sensitivity CAR-T cells against U87 cells with varied antigen density levels. Both high and low-sensitivity CAR-T cells showed similar cytotoxicity against high-antigen density U87 cells, while high sensitivity CAR-T cells exhibited stronger cytotoxicity against median and low-antigen density U87 cells (*n* = 4 independent samples). **d**–**f** Long-term killing (24 h) of high or low-sensitivity CAR-T cells against U87 cells with different antigen density levels. Still, low-sensitivity CAR-T cells demonstrated weak cytotoxicity against low-antigen density U87 cells. High and low-sensitivity CAR-T showed similar cytotoxicity against medium and high-antigen density U87 cells in the long-term killing assay (*n* = 4 independent samples). **g** Representative bioluminescent image of tumor burden of U87 cells with EGFRvIII low density, followed by the treatment with high (green) or low-sensitivity (red) CAR-T cells. NC, peripheral blood T cells. **h**, **i** Bioluminescence intensity (total flux) and Kaplan–Meier analysis of mice in **g**, *n* = 5–7 per group. **j** Representative bioluminescent image of tumor burden using U87 cells with EGFRvIII high density, followed by the treatment with high (red) or low-sensitivity (green) CAR-T cells. **k**, **l** Bioluminescence intensity (total flux) and Kaplan–Meier analysis of mice in **j**, *n* = 5–7 per group. Data are represented as mean ± SD, unpaired Student’s *t* test, *P < 0.05; **P < 0.01; ***P < 0.001, ****P < 0.0001

Previous results have indicated the contribution of trogocytosis to tumor therapeutic resistance, and the correlation between trogocytosis and cholesterol metabolism, as well as the translational potential of atorvastatin. However, a rapid regression of the tumor burden has not been observed to result from those combination therapy strategies (CAR-T Flag, or combining with atorvastatin). Further, based on the comprehensive clinical characteristics of patients, a majority of present ongoing clinical trials involve CAR-T monotherapy (NCT05577991, NCT02208362, and NCT02442297). In order to enhance the therapeutic effect of CAR-T cells and facilitate the clinical translation of the concept of trogocytosis, we focused on tuning the antigen-sensitivity of CAR-T cells to match the tumor antigen density according to previously mentioned results. We investigated the administration of low-sensitivity CAR-T cells in high-antigen density tumors and high sensitivity CAR-T cells in low-antigen density tumors. This approach aimed to balance trogocytosis-mediated tumor antigen escape and CAR-T efficacy. To investigated the feasibility of this strategy, we compared the cytolytic activity of CAR-T cells of high or low sensitivity. As expected, high sensitivity CAR-T cells effectively lysed tumor cells of high antigen density in short-term (4 h) coculture (Fig. 5a–c). Interestingly, although high sensitivity CAR-T cells exhibited greater killing effect in the early phase of coculture, low sensitivity CAR-T cells showed equal or better efficiency in long-term (24 h) coculture against U87 of high and medium antigen density (Fig. 5d–f). These in vitro experiments support the translational potential of this strategy of tuning the antigen-sensitivity of CAR-T cells to match the tumor antigen density.

To evaluate these findings in animal models, we tested high and low-sensitivity CAR-T cells in high and low-antigen density tumor models, respectively. U87 cells with either high or low expression of EGFRvIII were utilized for intracranial modeling of substantial tumor burdens. Mice received two doses of the corresponding CAR-T cells. We observed feeble response of low-sensitivity CAR-T cells against low-antigen density tumor models whereas high sensitivity CAR-T cells demonstrated potential effects and extended survival (Fig. 5g–i). In high-antigen density models, high sensitivity CAR-T cells were unable to effectively inhibit tumor recurrence, while low-sensitivity CAR-T cells promoted potential tumor regression (Fig. 5j–l). Similar results were replicated in LNCaP cells (Supplementary Fig. 7f), where PSMA-targeted CAR-T cells of low-sensitivity rapidly decreased the tumor burden. Collectively, these functional experiments suggest that optimizing CAR sensitivity to align with the tumor antigen density could enhance CAR-T therapy by circumventing trogocytosis.

## Discussion

The loss of target antigens represents a primary mechanism of immune evasion in response to CAR-T treatment. Beyond the numerous studies focusing on the long-term and permanent mechanisms of tumor therapeutic resistance,^58^ a growing body of evidence also indicates that short-term adaptations also contribute to the issue of resistance,^25,59,60^ and thus offering an opportunity for the transcriptomic or genomic alteration of tumors. Additionally, the dysfunction and modification of T cells have been detected within hours following their encounter with tumors.^61^ These previous works demonstrate that short-term immune modification and adaption may contribute to the consequence of tumor-immune cell interactions, highlighting the importance of such issue. In this study, our objective was to visualize and characterize one possible form of short-term resistance, that trogocytic CAR molecule transfer may contribute to antigen loss and CAR-T cell dysfunction.

Previous works have highlighted the widespread occurrence of trogocytosis in cellular interactions,^62,63^ identifying T, CAR-T cells, and other lymphocytes as potential acceptor cells in this process.^24,27,30,35,38,64,65^ Our findings herein reveal, the possibility of the role exchange in solid tumors, whereby CAR-T cells donate CAR molecules to tumor cells. A recent study, based on immunoelectron microscopy, highlights the phenomenon that T cell “budded” T cell receptor (TCR) via ectocytosis.^66^ We here shed light not only on the phenomenon that T cells can act as donor cells, but also offer another view differing from those held by peers in this area, who believe that the removal of antigen recognition area (TCR) facilitates detachment and serial killing. Our data instead suggests that the consequence of such molecule transfer at least reduces the CAR molecule density of CAR-T cell and dampens further cytotoxicity. This idea can be indirectly supported by another recent study, where nanotube-mediated mitochondrial trafficking metabolically benefits tumor evasion and impairs immune cell cytotoxicity.^67^ Meanwhile, the acquired CAR molecule may lead to antigen masking. This idea can be supported by the case report, where CAR-expressing leukemic B cell masked in cis to the CD19 epitope to avoid recognition.^68^ These two potential roles of trogocytic CAR molecule transfer, appear to contribute to short-term therapeutic resistance, thereby expanding the current understanding of trogocytosis-associated antigen escape.

We next sought to identify the underlying factors associated with CAR molecule transfer and to explore potential translational strategies to counteract this dynamic feature. Our study examined common factors related to CAR-T efficiency, ultimately focusing on the CAR-target relationship, including antigen density, effector-to-target ratio, and CAR molecule sensitivity, while excluding checkpoint and CAR downstream signaling activation. Intriguingly, our data indicate a link between CAR molecule structure, a determinant of CAR sensitivity, and trogocytosis. This finding advances those prior conclusions that trogocytosis is primarily dependent on antigen density and scFv sensitivity.^24,39^

Tumor cells acquire transient resistance to CAR-T therapy by trogocytosing CAR molecules, leading to tumor recurrence post-CAR-T infusion. The tumor recurrence post CAR-T infusion can be partially restrained by administering a salvage dose of anti-CAR CAR-T cells. To balance the potential fratricide effect with CAR-T cell kinetics, the salvage dose of the anti-CAR CAR-T cells is administered two weeks^69^ after the first infusion. The brisk regression of tumors following anti-CAR CAR-T administration indicates the possibility of trogocytic CAR molecule transfer in vivo.

We next focused on the underlying mechanism and its translational potential. Because cholesterol metabolism is a well-known factor in membrane biomechanics and protein interaction,^70–72^ and retrospective studies have reported the protection effect of statins against cancer,^73–75^ we hypothesized that inhibiting HMG-CoA reductase (the rate-limiting enzyme of the mevalonate pathway that is enriched in various kinds of malignancies)^76,77^ may protect CAR molecule from the undergoing transfer, thus facilitating serial killing. Similar to previous research,^50^ our data suggests that cholesterol regulation alleviates trogocytic CAR molecule transfer, thereby promoting the killing of tumor cells by CAR-T cells.

In view of the following points: 1. The combination therapy involving anti-CAR CAR-T cells or atorvastatin did not achieve rapid tumor regression, 2. several CAR-T clinical trials did not incorporate tags on the CAR molecules (NCT02664363, NCT02208362, NCT04185038, NCT05577091), and 3. the potential risks of anti-CAR CAR-T cells and the underlying fratricide effect remain unknown, we aimed to optimize the CAR-T cell structure for tumor eradication and propose a CAR-T monotherapy strategy for clinical translation, using the concept of CAR molecule trogocytosis.

Previous results and reports have suggested that CAR sensitivity is considered a key factor in therapeutic efficacy and is strongly related to trogocytosis.^43,44,78,79^ We propose that modulating CAR sensitivity is a potential and feasible strategy to balance trogocytosis-induced antigen escape and CAR-T cell-mediated cytotoxicity. Although tuning CAR sensitivity does not completely prevent trogocytosis, our data suggest that milder CAR sensitivity improves outcomes following consecutive CAR-T cell administration, potentially due to weaker trogocytosis. This finding may be indirectly supported by other existing animal experiments and clinical reports, demonstrating superior outcomes with lower sensitivity CAR-T cells.^80,81^ Considering the current clinical practice of administering multiple infusions with single-structure CAR-T cells^7,82^ and the lack of approved combination therapies involving multiple CAR-T cell structures or metabolic drugs, a more feasible approach may involve the administration of individualized CAR-T cells, that is low-sensitivity CAR-T cells targeting highly expressed antigens (e.g., B7-H3,^83^ GD2^84^) and high sensitivity CAR-T cells targeting modestly expressed antigens (e.g., IL13Ra, EGFRvIII). These strategies require pathological immunohistochemistry examination prior to CAR-T infusion and individualized CAR-T cells with antigen density-matched sensitivity.

In summary, our study reveals a previously unreported mechanism in which tumor cells trogocytose CAR molecules via CAR-T immune synapses, resulting in short-term antigen escape, CAR-T cell dysfunction, and ultimately promoting therapeutic resistance. This feature may be related to tumor cell cholesterol metabolism and can be mitigated by administering multiple infusions of CAR-T cells with antigen density-adapted sensitivity. A deeper understanding of this dynamic process may contribute to refining the clinical CAR-T therapy framework for solid tumors.

## Materials and methods

### Cells and culture conditions

Cells were maintained in DMEM/F12 supplemented with B27 (Gibco), human epidermal growth factor (hEGF, PeproTech, 20 ng/mL), and human fibroblast growth factor (hFGF, PeproTech, 20 ng/mL) for GSCs, or in DMEM containing 10% FBS and penicillin-streptomycin for U87 cell lines, or in RPMI-1640 supplemented with 10% FBS and penicillin-streptomycin for LNCaP cell lines. Cell lines were transduced with either pLV-HSV-TK promoter-hluciferase-hef1a-eGFP-P2A-neo or pLV-HSV-TK promoter-hluciferase-hef1a-mScarlet-P2A-neo lentivirus and subsequently sorted under G-418 selection (400 μg/mL, Solarbio) for a minimum of two weeks. To generate different EGFRvIII densities, luciferase-eGFP^+^ U87 cells underwent fluorescence-activated cell sorting following transduction with the pLV-HSV-TK promoter-EGFRvIII-puro lentivirus.^44^ Concurrently, pLV-HSV-TK promoter-EGFRvIII-eGFP-puro lentivirus was transduced into U87 cells under puromycin selection (0.5–5 μg/mL, Solarbio) for at least two weeks.

### Plasmids and lentivirus production

The CAR constructs used in this study were based on adeno-associated virus (AAV)-derived vectors containing a CD8α leader sequence, a CD8 hinge with or without CH2CH3 domain, a CD28 transmembrane and intracellular domain, and a CD3ζ domain. The scFvs of anti-EGFRvIII, anti-B7-H3, anti-PSMA, or anti-Flag-tag antibodies were utilized to generate corresponding CAR genes. ScFvs targeting EGFRvIII, B7-H3, and PSMA were labeled with a FLAG-tag at the 5’ end of their respective sequences. To trace CAR molecules, an anti-EGFRvIII-CD8 hinge-CD28 transmembrane-CD28 intracellular-CD3ζ-linker-mScarlet, or anti-EGFRvIII-CD8 hinge-CD28 transmembrane-CD28 intracellular-CD3ζ-linker-miRFP670nano3^85^ CAR sequence was constructed. The full-length EGFRvIII DNA was fused with an eGFP sequence at its 3’ end as required. Lentiviral products were obtained from Beijing SyngenTech Co., Ltd.

### CAR-T cell production

Peripheral blood T cells were activated using T Cell TransAct™ (Miltenyi Biotec) according to the manufacturer’s instructions. CAR-encoding lentiviruses were mixed and added to Retronectin (Takara)-precoated culture plates, followed by centrifugation at 2000g for 90 min and supplementation with 5 μg/mL polybrene. Cells were cultured in ImmunoCult™-XF T Cell Expansion Medium (StemCell) containing 5 ng/mL IL-15 (PeproTech) and 10 ng/mL IL-7 (PeproTech).

### Antibodies and flow cytometry assays

CAR-T cells were pre-stained with PKH67 or MINCLARET (Sigma Aldrich) as per the manufacturer’s guidelines. To induce PD1 expression in the CAR-T cells, they were cultured in conditioned medium (consisting of the supernatant collected from CAR-T and tumor cell cocultures), two days prior to subsequent experiments. Tumor cells and CAR-T cells were cocultured at an effector-to-target ratio of 1:1 overnight, and then subjected to thorough digestion using Accutase (Gibco) and cell pellet filtration to obtain a single cell suspension. For the cell dye group, cells were stained and cocultured, then directly analyzed using a flow cytometer (BD C6 Plus). For molecular detection, cells were fixed and incubated with antibodies for 20 min at room temperature. Atorvastatin (5 μM, MCE) or bezafibrate (50 μM, MCE)was added 3 days prior to CAR-T cell engagement. Cytochalasin B (10 μg/mL, MCE) was added 1 day and discarded prior to CAR-T cell engagement. CAR-T cells were pre-treated with ZAP180013 (5 μM, MCE) for 1 h before tumor engaging, as needed. Cells were then stained with monoclonal antibodies against FLAG-tag (APC, BioLegend), PD1 (PE, BioLegend), EGFRvIII (Invitrogen), and anti-rabbit IgG (Alexa Fluor 647, CST). For antigen masking assessment, the recombinant scFv (fused with myc-tag, 1 μg/mL) of EGFRvIII-CAR molecule was used. For in vivo trogocytosis assessment, animals with substantial intracranial tumor models were administered with 10^6^ CAR-T cells. Tumor tissues were harvested at indicated time points and digested with Tumor Dissociation Kit (Miltenyi Biotec). Tumor cells were located by FSC-A, SSC-A and FITC fluorescence values. Flow cytometric data were analyzed using FlowJo v10 software.

### Immunoblotting assays

For T cell and tumor cell isolation, surplus single cell suspension was filtered with cell strainer (pluriSelect,10 μm) to preliminarily separate T cell and tumor cell by size. The cells were then magnetically purified with a T cell positive selection kit (Invitrogen) to reserve or remove T cells, as needed. For the phosphorylation test, CAR-T and tumor cells were resuspended in PBS at an E: T = 1:1. The cells were harvested and lysed at indicated time points. CAR-T and tumor cells were cocultured at an E: T = 1:1 overnight to produce Trogo^+^ tumor and TrogoED CAR-T cells. The cell mixtures were then isolated for further coculture and phosphorylation tests. Anti-FLAG tag antibody (Proteintech) was used for CAR molecule detection.

### Killing assays

Two days before the killing assays, target cells (luciferase-eGFP labeled) were induced to adhere by culturing them in poly-L-lysine coated plates with corresponding medium. Tumor cells were the cocultured with CAR-T cells for 4 h or 24 h at indicated effector-to-target ratios. Subsequently, luciferase substrate (PerkinElmer) was added to each well, and the emitted light was detected using the IVIS Spectrum In Vivo Imaging System (PerkinElmer). Maximal luminescence was determined using target cells alone (Avg_conctol_). Lysis was calculated as (1 − Avg_sample_/Avg_conctol_) × 100%.

### Electron microscopy

Adherent EGFRvIII^+^ U87 cells were cocultured with corresponding CAR-T cells overnight. The CAR-T cells were then removed along with the supernatant. The remaining cells were scraped and fixed in 2.5% glutaraldehyde at room temperature. Subsequently, cells were stained with colloidal gold-labeled anti-FLAG-tag antibody overnight, contrasted with 1% osmium acid after gradient dehydration, and imaged using the Hitachi TEM system (HT7800).

### Immunofluorescence and confocal microscopy

GFP-tagged tumor cells were adhered to poly-L-lysine precoated glass-bottom dishes prior to the addition of CAR-T cells. For cell dye microscopy, CAR-T cells were pre-stained with PKH26 (Sigma Aldrich) following the manufacturer’s instructions. The cells were then cocultured overnight and fixed. For direct observation, cell dye microscopy samples were fixed and mounted with DAPI (Solarbio). For molecular detection, samples were stained with primary antibodies against FLAG-tag (Proteintech) at a dilution of 1:200 for two hours at room temperature. Alexa Fluor 647-conjugated secondary antibodies (1:500, Abcam) were used to detect primary antibodies. Samples were counterstained with DAPI. CD3 was detected with CoraLite594-conjugated primary antibody (Proteintech). Mounted samples were imaged using microscopy (Observer Z1 and Axio Scan Z1, ZEISS).

### CAR molecule transfer and microscopic tracing

To perform cell dye tracing, EGFRvIII-targeted CAR-T cells were pre-stained with PKH67 (Sigma Aldrich) and cocultured with adherent EGFRvIII-positive U87 cells overexpressing mScarlet. For CAR molecule tracing, T cells expressing the EGFRvIII scFv-CD28-CD3ζ-mScarlet fusion protein were cocultured with U87 cells overexpressing the EGFRvIII-GFP fusion protein. The cell mixture was placed in a confocal dish and observed using a Live Cell Station (Zeiss Axio Observer Z1). Images were captured every 3–5 min.

### Mice

All mice were maintained under specific pathogen-free conditions in a barrier facility at Beijing Tiantan Hospital. All animal care, monitoring, and experimentation were conducted in accordance with and approved by the Beijing Tiantan Hospital Laboratory Animal Care Ethics Committee.

### Orthotopic xenograft model for brain tumors

EGFP-luciferase-tagged GSCs and U87 cells (with either high or low EGFRvIII expression) were orthotopically injected into the frontal lobe of 5-week-old NOD.Cg-Prkdc^scid^IL2r^gtm1Vst^/Vst female mice (obtained from Vitalstar Biotechnology Co., Ltd., Beijing). Mice were then anesthetized using 3% isoflurane. A burr hole was created 2 mm lateral and 1 mm anterior to the bregma. A blunt-ended needle (Hamilton Company) was inserted into the burr hole to a depth of 3.5 mm below the dura. Using a microinjection pump, 4 ×10^5^ cells were suspended in PBS and injected in a volume of 5 μL over 3 min. Tumor growth was monitored via bioluminescent imaging (BLI) using an IVIS Spectrum In Vivo Imaging System (PerkinElmer) and quantified with the Live Image v.4.0 software (PerkinElmer). The primary locoregional infusion of CAR-T or peripheral blood T cells were processed when the total flux[p/s] reached 10^7^. Atorvastatin (40 mg/kg/day by oral gavage) administration began 3 days prior to the first dose of CAR-T cells, and continued until one week post the infusion of the second dose of CAR-T cells.

### Subcutaneous models for prostate tumors

LNCaP cells were implanted subcutaneously in the inter-scapular space in 5-week-old NOD.Cg-Prkdc^scid^IL2r^gtm1Vst^/Vst male mice (10^6^ cells in 100 μL PBS per mouse). CAR-T or peripheral blood T cells (10^6^ CAR-T cells in 100 μL PBS per mouse) were intravenously administered when the tumor volume reached 100 mm.^3^ Atorvastatin administration was conducted in the same manner as with the GBM models.

### Statistical analysis

Significance of differences between the two groups was determined using the unpaired Student’s *t* test (*p < 0.05, **p < 0.01 ***p < 0.001, ****p < 0.0001). Log-rank (Mantel–Cox) test was used for comparison of survival curves. All data presentation and statistical analyses in the article were conducted using GraphPad (version 8.0) and R (version 4.0.0, http://www.r-project.org). A *p*-value of less than 0.05 was considered to indicate statistically significant differences. All statistical tests were two-sided.

## Limitations of this study

In addition to the phenomenon of trogocytosis (antigen and CAR molecule transfer), CAR molecule degration^11^ and, antigen depletion^26^ may also be involved in the dynamic and short-term modulation of membrane molecules. Further investigation with respect to their dynamic balance and the underlying mechanisms is needed. In addition, our data suggests that atorvastatin inhibits trogocytosis and improves therapeutic effect of CAR-T cells. There is lack of evidence that atorvastatin enhances CAR-T efficacy mainly by inhibiting trogocytosis in vivo. Meanwhile, the underlying biochemical mechanism of atorvastatin inhibiting trogocytosis needs further investigation.

### Supplementary information

Movie S1. CAR-T cell membrane was transferred to target cells via immune synapse

Movie S2. CAR molecule was transferred to target cells via immune synapse

Movie S3. Three-dimensional reconstruction of confocal microscopy of CAR-T against LNCaP cell

Supplementary Materials

All original films of Western blots

## Data Availability

The data used in this study are available from the corresponding authors upon reasonable requests.
