# Supplementary material for: Trogocytosis of CAR molecule regulates CAR-T cell dysfunction and tumor antigen escape
Source: Signal Transduct Target Ther. 2023 Dec 25;8:457. doi: 10.1038/s41392-023-01708-w (PMC10749292; doi:10.1038/s41392-023-01708-w)
Supplement: Supplementary file 4 — Supplementary Materials [file 41392_2023_1708_MOESM4_ESM.docx]

Supplementary Materials for

Trogocytosis of CAR molecule regulates CAR-T cell dysfunction and tumor antigen escape

You Zhai, Yicong Du, Guanzhang Li, Mingchen Yu, Huimin Hu, Changqing Pan, Di Wang, Zhongfang Shi, Xu Yan, Xuesong Li, Tao Jiang, Wei Zhang

Correspondence to: Wei Zhang zhangwei_vincent@mail.ccmu.edu.cn，and Tao Jiang taojiang1964@163.com

**This PDF file includes:**

Supplementary Figure legends

Figures. S1 to S7

Table S1

Captions for Movies S1 to S3

**Other Supplementary Materials for this manuscript include the following:**

Movies S1 to S3


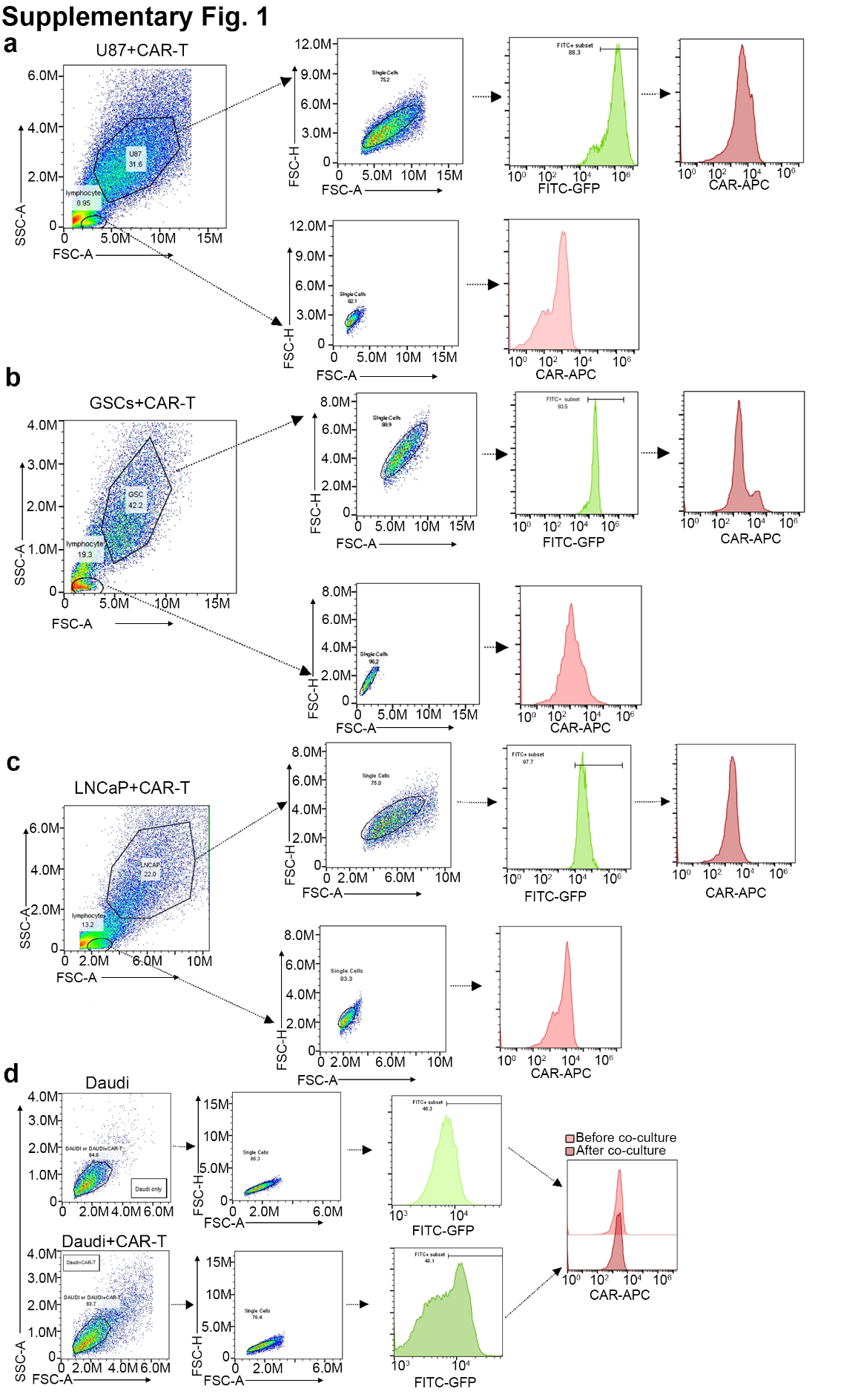


Figure. S1. Gating strategy and trogocytosis.

**(a–c)** GFP-labeled tumor cells (U87, GSC, LNCaP) were cocultured with corresponding CAR-T cells. Tumor and CAR-T cell colonies were preliminarily gated and separated using FSC-A and SSC-A values. Tumor cells were further gated based on single-cell colonies and FITC fluorescence. A Flag-tag was inserted at the N'-terminal of the CAR molecule and was detected with APC-labeled anti-FLAG-tag antibody. **(d)** GFP-labeled CD19^+^ DAUDI cells alone or cocultured with CD19-targeting CAR-T cells were assessed by flow cytometry. No significant CAR molecule acquisition was detected (n = 6 independent duplicates).


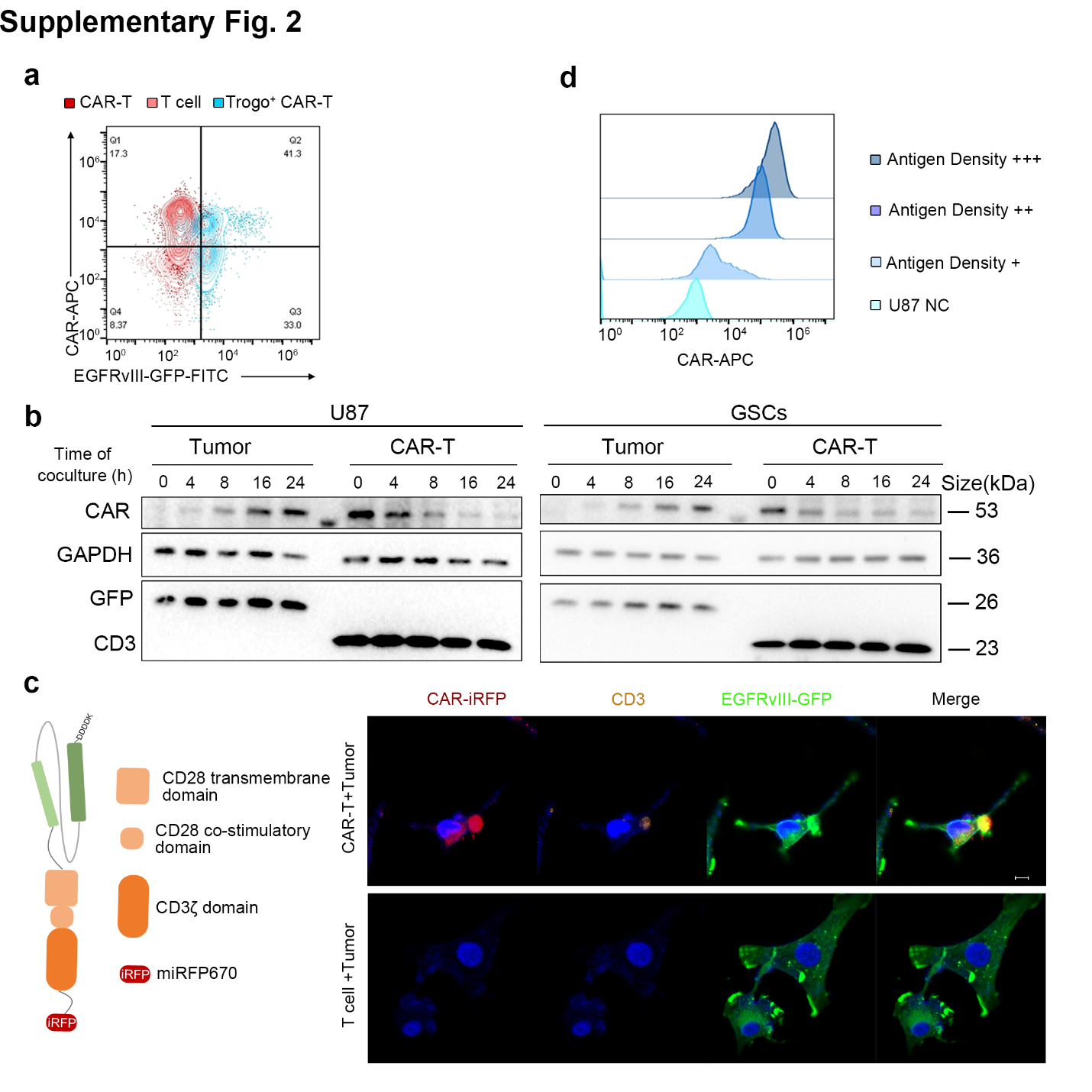


Figure. S2. Transfer of CAR molecule and target antigen.

**(a)** EGFRvIII-targeted CAR-T cells alone or cocultured with U87 cells (EGFRvIII-eGFP fusion protein-overexpressed) were assessed by flow cytometry. Gate on CAR-T or T cells. The result suggests that EGFRvIII-eGFP fusion protein may transfer to CAR-T cells (Trogo^+^ CAR-T, blue), consistent with the previous reports (n = 6 independent duplicates). **(b)** CAR molecule acquiring of tumor cells and loss of CAR-T cells increased with time. EGFRvIII-targeted and B7-H3-targeted CAR-T cells were respectively cocultured with GFP-labeled tumor cells (U87 and GSCs). Cells were isolated and assessed at indicated coculture time points. Immunoblotting images are represented among at least three independent duplications. **(c)** Schematic structure of CAR molecule for cell tracing (Left). EGFRvIII-eGFP fusion protein overexpressed U87 cells were cocultured with the corresponding CAR-T cells or peripheral blood T cells. Representative images show that miRFP-labeled CAR molecules (deep red) were trogocytosed to tumor cells (green), while CD3 (orange) was not (Right, scale bar: 5 μm). **(d)** Different expression levels of EGFRvIII in U87 cells. Cells were detected and sorted by using EGFRvIII primary antibody, followed by APC-labeled secondary antibody.


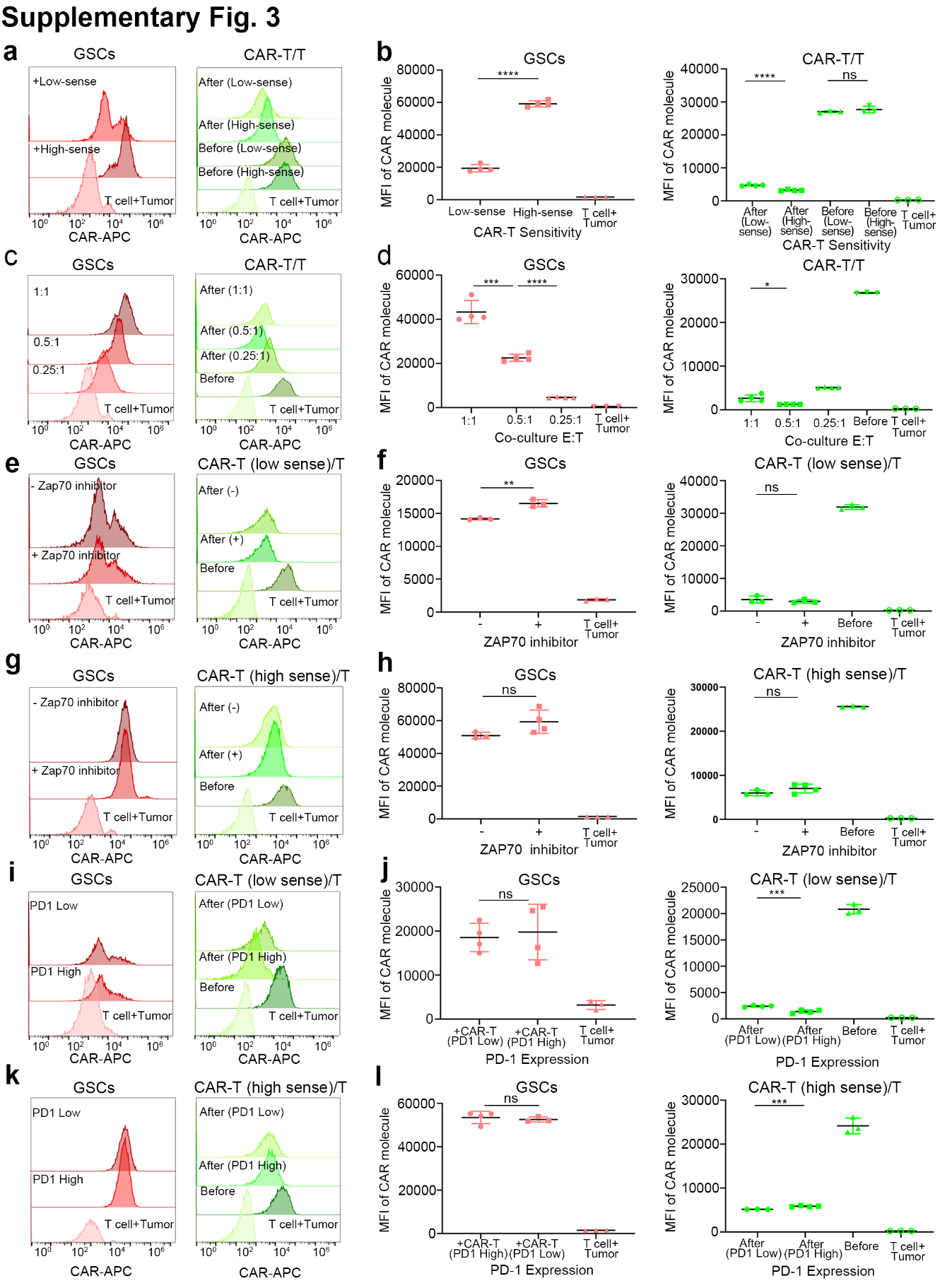


Figure. S3. Trogocytic CAR molecule transfer was also detected in GSCs with the same tendency of U87 cells.

**(a, b)** GSCs were cocultured with high sensitivity (High-sense, B7-H3 scFv-CD28-CD3ζ) or low-sensitivity (Low-sense, B7-H3 scFv-CH2CH3-CD28-CD3ζ) B7-H3-targeted CAR-T cells. Data indicate that CAR molecule transfer depends on its sensitivity. More replicates and statistical differences are shown in (b). **(c, d)** GSCs were cocultured with high sensitivity CAR-T cells at specified E: T ratios. Consistent with the results in U87 cells, CAR molecule trogocytosis is correlated with E: T. Independent replicates and statistical differences are shown in (d). **(e, f)** GSCs were cocultured with low-sensitivity CAR-T cells, with (+) or without (-) the supplement of ZAP70 inhibitor. No biological difference was observed between the two groups. Independent replicates and statistical differences are shown in (f). **(g, h)** GSCs were cocultured with high sensitivity CAR-T cells. The result displays similar to (e) and (f). **(i–l)** B7-H3 CAR-T cells of low-sensitivity (i) and high sensitivity (k) were pre-induced to express PD1 before coculture with GSCs. Similar to those of U87 cells, data indicate that trogocytic CAR molecule transfer is independent of PD1 expression in GSCs. Additional replicates are provided in (j and l). Data are represented as mean ± SD, unpaired Student’s t-test, *P < 0.05; **P < 0.01; ***P < 0.001, ****P < 0.0001.


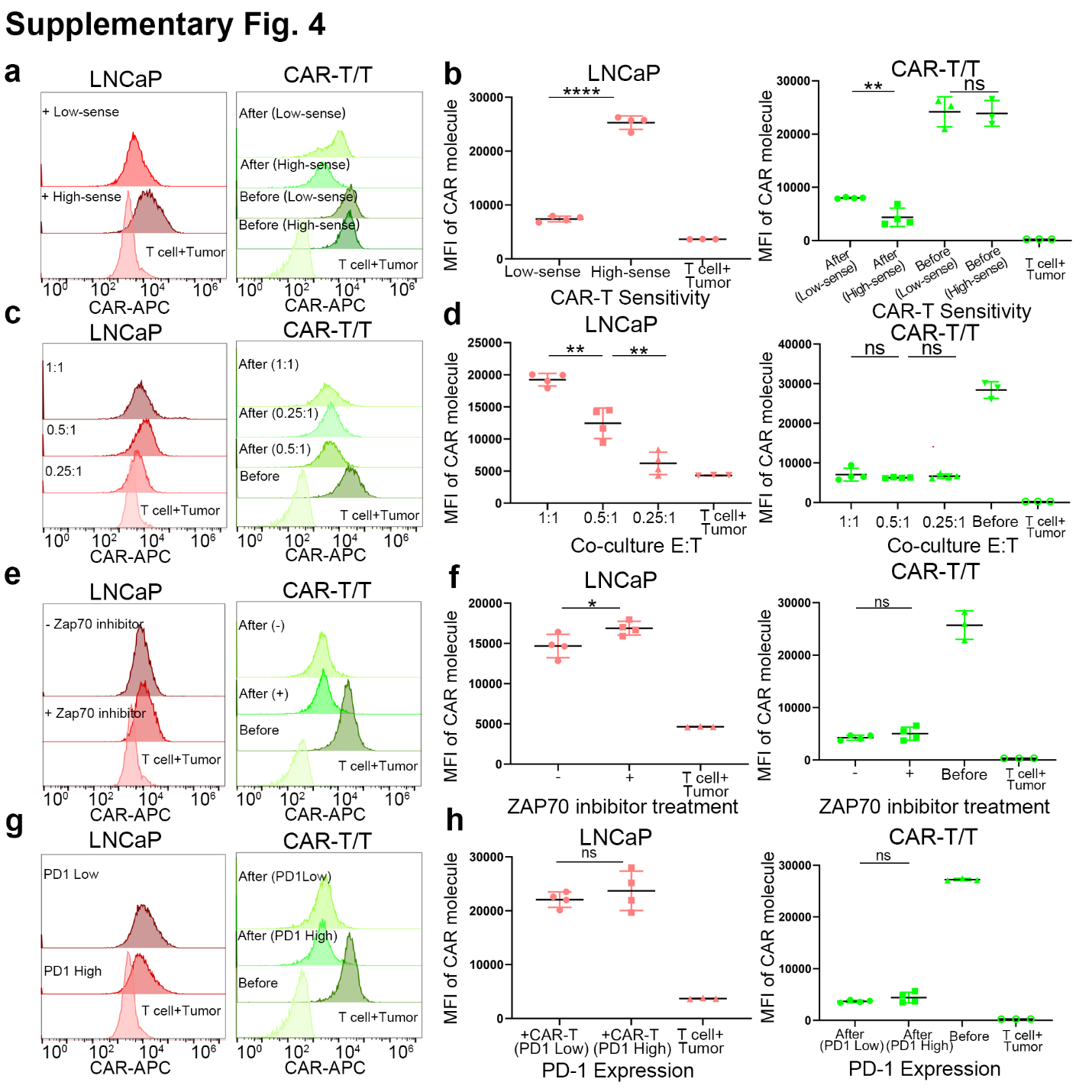


Figure. S4. CAR molecule trogocytosis was observed in prostate tumor, consistent with glioblastoma.

**(a, b)** LNCaP cells were cocultured with high sensitivity (High-sense, PSMA scFv-CD28-CD3ζ) or low-sensitivity (Low-sense, PSMA scFv-CH2CH3-CD28-CD3ζ) PSMA-targeted CAR-T cells. The data support the notion that CAR molecule transfer is correlated with CAR molecule sensitivity. Additional replicates and statistical differences are shown in (b). **(c, d)** LNCaP cells were cocultured with high sensitivity PSMA CAR-T cells at indicated E: T ratios. Consistent with glioblastoma, the result shows that trogocytosis is correlated with E: T ratio. Independent replicates and statistical differences are shown in (d). **(e, f)** LNCaP cells were cocultured with high sensitivity CAR-T cells with (+) or without (-) the supplement of ZAP70 inhibitor. No biological difference was observed between the two groups. Independent replicates and statistical differences are shown in (f). **(g, h)** CAR-T cells (PSMA-targeted, high sensitivity) were pre-induced to express PD1 before coculture with LNCAP cells. Data support the idea that trogocytic CAR molecule transfer is independent of PD1 expression. Additional replicates and statistical differences are provided in (h). Data are represented as mean ± SD, unpaired Student’s t-test, *P < 0.05; **P < 0.01; ***P < 0.001, ****P < 0.0001.


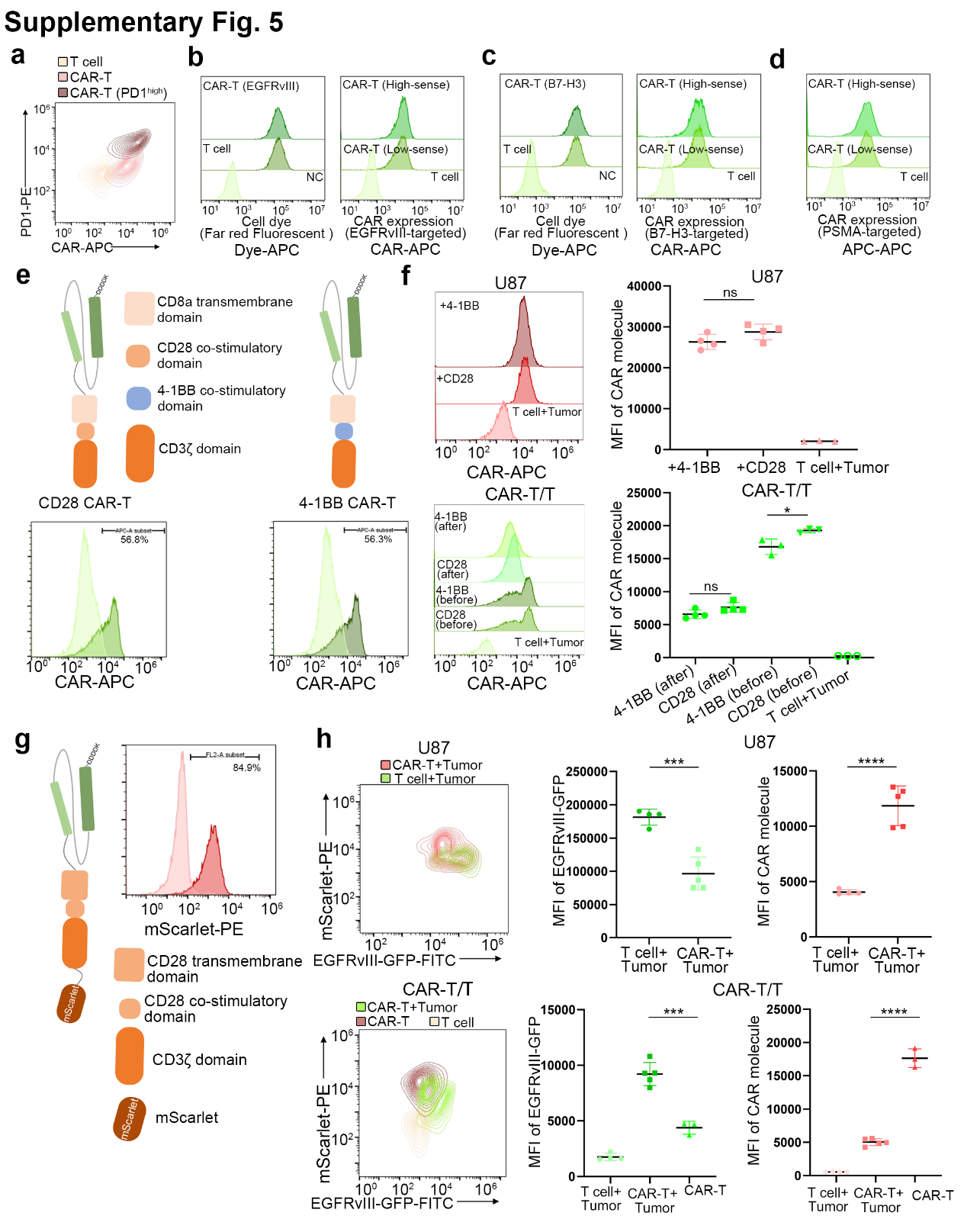


Figure. S5. CAR-T cell immunophenotype and other possible factors of trogocytosis.

**(a)** PD1 was induced to express in EGFRvIII-targeted CAR-T cells via conditioned medium. Data are from at least three independent experiments. **(b)** Left, deep red fluoresces of EGFRvIII-targeted CAR-T cells or T cells before coculture with U87 (EGFRvIII overexpressed). Right, the transduction efficiency of EGFRvIII-targeted CAR-T cells (high and low sensitivity). Data are from at least three independent experiments. **(c)** Left, deep red fluoresces of B7-H3-targeted CAR-T cells or T cells before coculture with GSCs. Right, the transduction efficiency of B7-H3-targeted CAR-T cells (high and low sensitivity). Data are from at least three independent experiments. **(d)** Transduction efficiency of PSMA-targeted CAR-T cells (high and low sensitivity). Data are from at least three independent experiments. **(e)** Schematic diagram of newly constructed CAR molecule and the transduction efficiency. CD28 or 4-1BB co-stimulatory domains were introduced into the CAR molecule. CD8a transmembrane domain was used to replace the CD28 transmembrane domain to minimize the synergistic effect with the CD28 co-stimulatory domain. Representative diagrams are from at least three independent experiments. **(f)** Left, U87 cells of EGFRvIII expression (high antigen density) were cocultured with CAR-T cells of different co-stimulatory domains overnight. Data indicate that CAR molecule transfer is not dependent on the choice of co-stimulatory domain. Right, independent duplications and statistical differences. **(g)** Schematic diagram of the CAR molecule with mScarlet and flow cytometric analysis of CAR surface expression. **(h)** Left, U87 cells (EGFRvIII-GFP overexpression) were cocultured with CAR-T cells overnight. Despite fluorescence leakage between FITC and PE, data indicate the exchange of CAR molecule and antigen between the effector and target cells. Right, independent duplications and statistical differences. Data are represented as mean ± SD, unpaired Student’s t-test, *P < 0.05; **P < 0.01; ***P < 0.001, ****P < 0.0001.


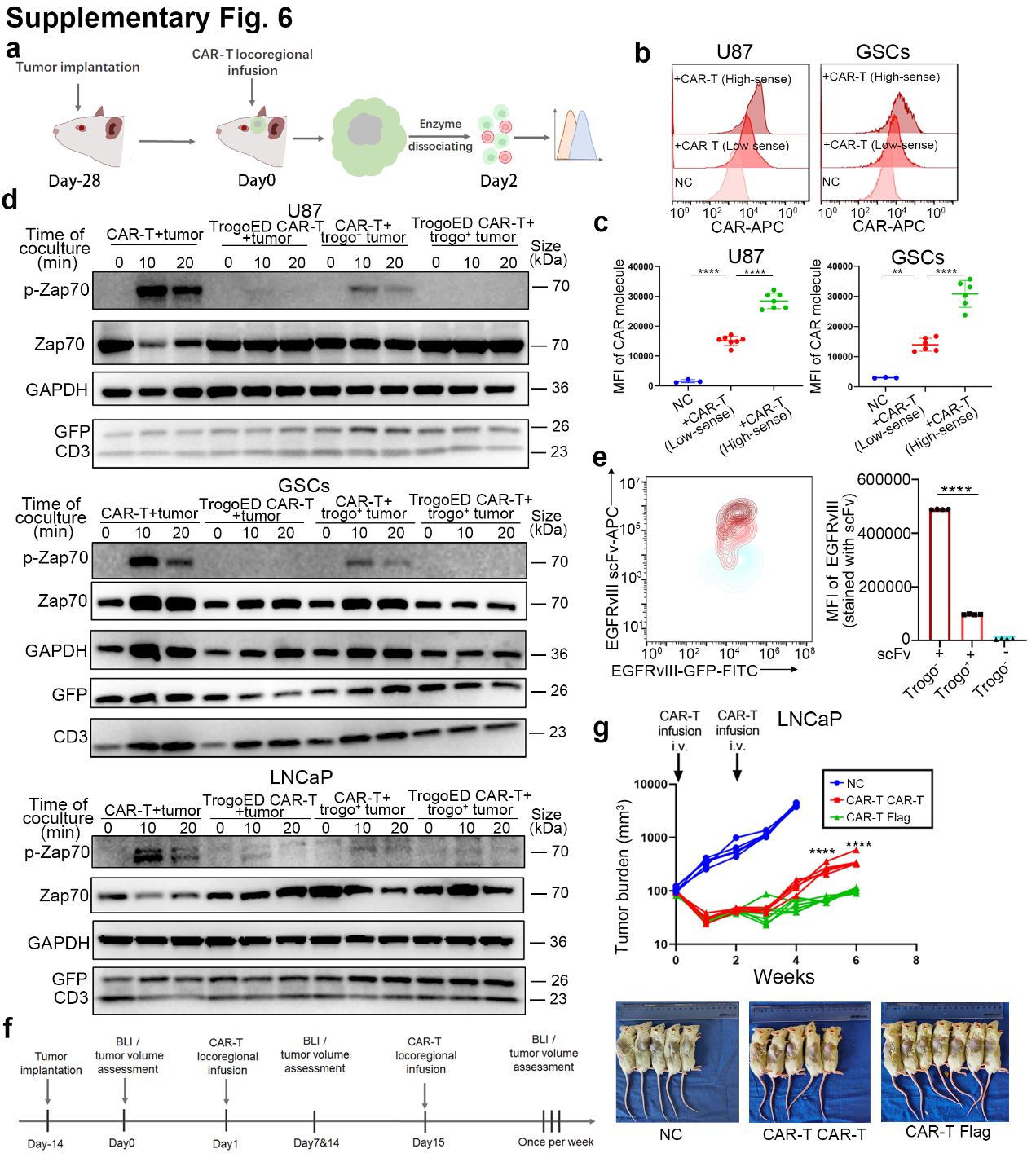


Figure. S6. *In vivo* experiments indicate the existence of CAR molecule trogocytosis.

**(a)** Schematic workflow of CAR molecule detection in intracranial GBM tissue post-CAR-T therapy. U87 (EGFRvIII high density) cells and GSCs were used. **(b, c)** Flow cytometric analysis of CAR molecule acquisition, gate on GBM tumor cells (U87 cells or GSCs, respectively). Consistent with in vitro experiments, tumor cells acquired more CAR molecules under the treatment of high sensitivity CAR-T cells. More duplications are shown in (c). N = 3 in the NC (peripheral blood T cells) group, n = 6-7 in CAR-T treatment group. **(d)** Fresh CAR-T cells or TrogoED CAR-T cells were cocultured with fresh or pre-cocultured (trogo^+^) tumor cells, respectively. The cell mixture was lysed at indicated time points. Phosphorylated ZAP70 levels in effector cells were evaluated by immunoblotting. Images are represented among three independent duplications. **(e)** Antigen masking of cocultured U87 cells (EGFRvIII-GFP overexpressed) was assessed with the scFv from EGFRvIII-targeted CAR-T cells. Independent duplications and statistical differences are shown on the right. **(f)** Schematic workflow of *in vivo* CAR-T treatment. Tumor-burdened animals were treated with EGFRvIII, B7-H3, and PSMA-targeted CAR-T cells according to tumor cell modeling, followed by primary CAR-T cells or FLAG-tag target CAR-T cells treatment. Bioluminescence or tumor volume intensity of tumor burden was assessed weekly. **(g)** Subcutaneous tumor models of LNCaP cells underwent intravenous CAR-T treatment. NC, peripheral blood T cells, n = 5 (blue); CAR-T CAR-T, two consecutive doses of PSMA-targeted CAR-T, n = 5 (red); CAR-T Flag, one dose of PSMA-targeted CAR-T followed by one dose of FLAG-tag targeted CAR-T, n = 7 (green). Tumors of each group at week 4 post the first dose of CAR-T cells are shown in the photos below. Data are represented as mean ± SD, unpaired Student’s t-test, *P < 0.05; **P < 0.01; ***P < 0.001, ****P < 0.0001.


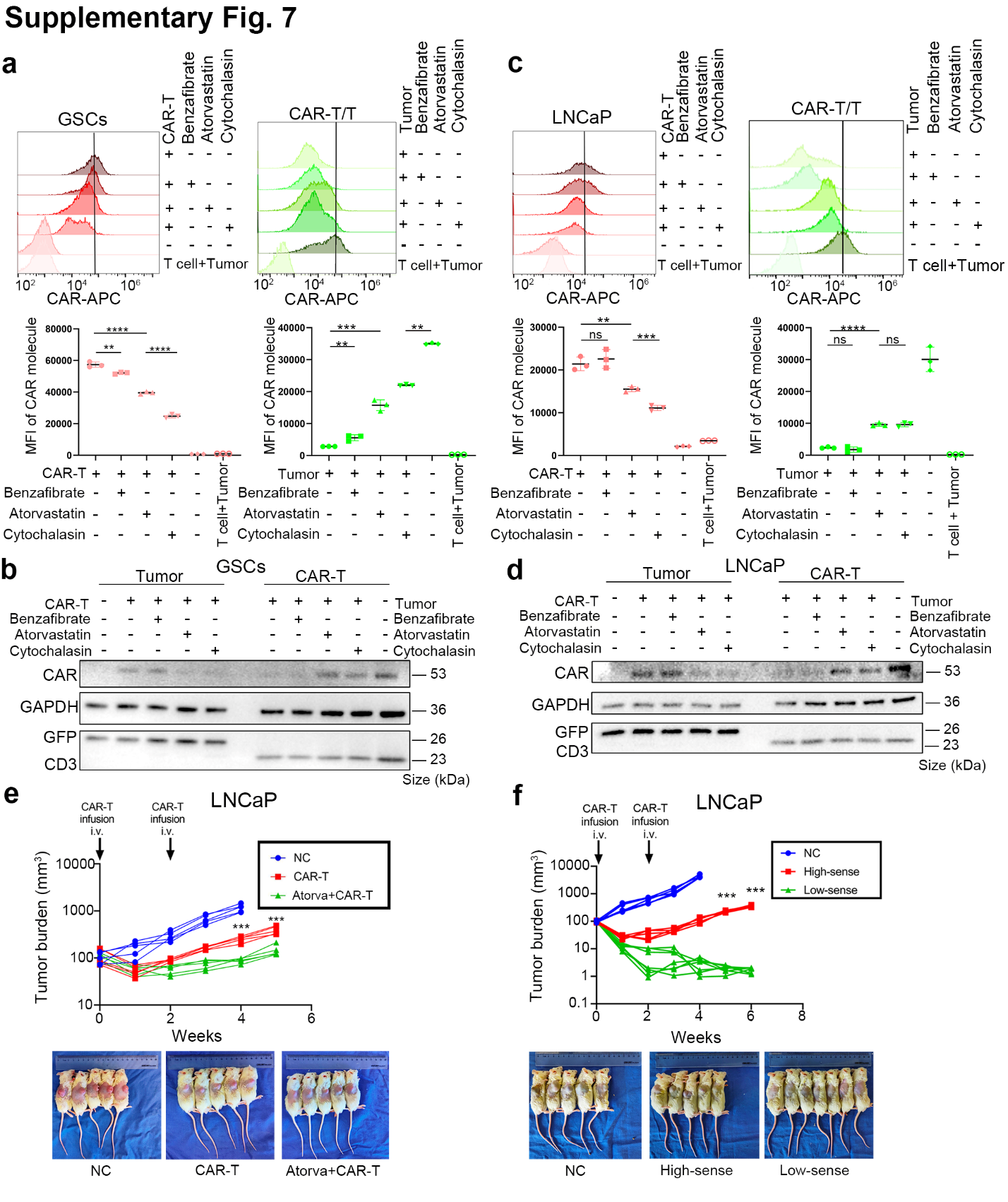


Figure. S7. The favorable effect of cholesterol inhibition on trogocytosis was also observed in other tumor models.

**(a)** GSCs were treated with B7-H3-targeted CAR-T cells (high sensitivity) or T cells, supplemented with indicated drugs. Gate on GSCs (left) and CAR-T cells (right). Flow cytometric data indicate that both atorvastatin and cytochalasin alleviated the CAR molecule acquisition of tumor cells and the CAR molecule loss of CAR-T cells. Additional replicates and statistical differences are provided below. **(b)** GSCs were cocultured with B7-H3-targeted CAR-T cells, supplemented with indicated drugs. Cells were magnetically isolated for CAR molecule detection. Consistent with relative results in U87 cells, representative immunoblotting image from three independent replications shows that cholesterol inhibition alleviates trogocytosis. **(c, d)** The same trend of the beneficial effect of atorvastatin was replicated in LNCaP cells. Data and images are from two to three duplications. **(e)** Subcutaneous tumor models of LNCaP cells were intravenously treated by PSMA-targeted CAR-T cells (high sensitivity). Atorvastatin was orally supplemented. N = 5 for each group. NC, peripheral blood T cells. Tumors of each group at week 4 post the first dose of CAR-T infusion are shown below. **(f)** Subcutaneous tumor models were intravenously treated by high or low sensitivity PSMA-targeted CAR-T cells. N = 5 in the NC (peripheral blood T cells) group (blue), n = 5 in the High-sense group (red), and n = 7 in the Low-sense group (green). Tumors of each group at week 4 post the first infusion are shown below. Data are represented as mean ± SD, unpaired Student’s t-test, *P < 0.05; **P < 0.01; ***P < 0.001, ****P < 0.0001.

Table S1. Protein amino sequences of CAR constructs

| scFv (MR1)  EGFRvIII target | QVQVLESGGGLVQPGGSLRLSCAASGFTFSSYAMSWVRQAPGKGLEWVSAISGSGGSTNYADSVKGRFTISRDNSKNTLYLQMNSLRAEDTAVYYCAGSSGWSEYWGQGTLVTVSSGGGGSGGGGSGGGGSDIQMTQSPSSLSASVGDRVTITCRASQGIRNNLAWYQQKPGKAPKRLIYAASNLQSGVPSRFTGSGSGTEFTLIVSSLQPEDFATYYCLQHHSYPLTSGGGTKVEIK |
| --- | --- |
| scFv (8H9)  B7H3 target | QVKLQQSGAELVKPGASVKLSCKASGYTFTNYDINWVRQRPEQGLEWIGWIFPGDGSTQYNEKFKGKATLTTDTSSSTAYMQLSRLTSEDSAVYFCARQTTATWFAYWGQGTTVTVSSDGGGSGGGGSGGGGSDIELTQSPTTLSVTPGDRVSLSCRASQSISDYLHWYQQKSHESPRLLIKYASQSISGIPSRFSGSGSGSDFTLSINSVEPEDVGVYYCQNGHSFPLTFGAGTKLELKQAAA |
| scFv (J591)  PSMA target | EVQLVQSGPEVKKPGATVKISCKTSGYTFTEYTIHWVKQAPGKGLEWIGNINPNNGGTTYNQKFEDKATLTVDKSTDTAYMELSSLRSEDTAVYYCAAGWNFDYWGQGTLLTVSSGGGGSGGGGSGGGGSDIQMTQSPSSLSTSVGDRVTLTCKASQDVGTAVDWYQQKPGPSPKLLIYWASTRHTGIPSRFSGSGSGTDFTLTISSLQPEDFADYYCQQYNSYPLTFGPGTKVDIK |
| scFv  CD19 target | QVQLQESGPGLVKPSETLSLTCTVSGVSLPDYGVSWIRQPPGKGLEWIGVIWGSETTYYSSSLKSRVTISKDNSKNQVSLKLSSVTAADTAVYYCAKHYYYGGSYAMDYWGQGTLVTVSSGGGGSGGGGSGGGGSEIVMTQSPATLSLSPGERATLSCRASQDISKYLNWYQQKPGQAPRLLIYHTSRLHSGIPARFSGSGSGTDYTLTISSLQPEDFAVYFCQQGNTLPYTFGQGTKLEIK |
| CH2CH3 | PSVFPLAPSSKSTSGGTAALGCLVKDYFPEPVTVSWNSGALTSGVHTFPAVLQSSGLYSLSSVVTVPSSSLGTQTYICNVNHKPSNTKVDKKVEPKSCDKTHTCPPCPAPELLGGPSVFLFPPKPKDTLMISRTPEVTCVVVDVSHEDPEVKFNWYVDGVEVHNAKTKPREEQYNSTYRVVSVLTVLHQDWLNGKEYKCKVSNKALPAPIEKTIS |
| CD8 hinge | TTTPAPRPPTPAPTIASQPLSLRPEACRPAAGGAVHTRGLDFACD |
| CD28 transmembrane | FWVLVVVGGVLACYSLLVTVAFIIFWV |
| CD8 transmembrane | IYIWAPLAGTCGVLLLSLVITLYC |
| CD28  co-stimulatory | RSKRSRLLHSDYMNMTPRRPGPTRKHYQPYAPPRDFAAYRS |
| 4-1BB  co-stimulatory | KRGRKKLLYIFKQPFMRPVQTTQEEDGCSCRFPEEEEGGCEL |
| CD3ζ | RVKFSRSADAPAYKQGQNQLYNELNLGRREEYDVLDKRRGRDPEMGGKPRRKNPQEGLYNELQKDKMAEAYSEIGMKGERRRGKGHDGLYQGLSTATKDTYDALHMQALPPR |

Movie S1. CAR-T cell membrane was transferred to target cells via immune synapse.

Green fluorescence-stained EGFRvIII-target CAR-T cells were cocultured with U87 cells (EGFRvIII overexpressed, red). Compartment of the green-stained CAR-T cell membrane gradually transferred to the U87 cell (scale bar: 5 μm).

Movie S2. CAR molecule was transferred to target cells via immune synapse.

CAR-T cells (EGFRvIII scFv-CD28-CD3ζ-mScarlet overexpressed, red) were cocultured with U87 cells (EGFRvIII-eGFP overexpressed, green). Granular CAR molecules (red) of the CAR-T cell transferred to the U87 cell (green) (scale bar: 5 μm).

Movie S3. Three-dimensional reconstruction of confocal microscopy of CAR-T against LNCaP cell.

PSMA-target CAR-T cells were cocultured with GFP-labeled LNCaP cells. CAR molecules (red) were observed to be trogocytosed to the cell membrane of the LNCaP cell (green).
