# Supplementary figures and images for: Trogocytosis of CAR molecule regulates CAR-T cell dysfunction and tumor antigen escape
Source: Signal Transduct Target Ther. 2023 Dec 25;8:457. doi: 10.1038/s41392-023-01708-w (PMC10749292; doi:10.1038/s41392-023-01708-w)

**All original films of Western blots**

**
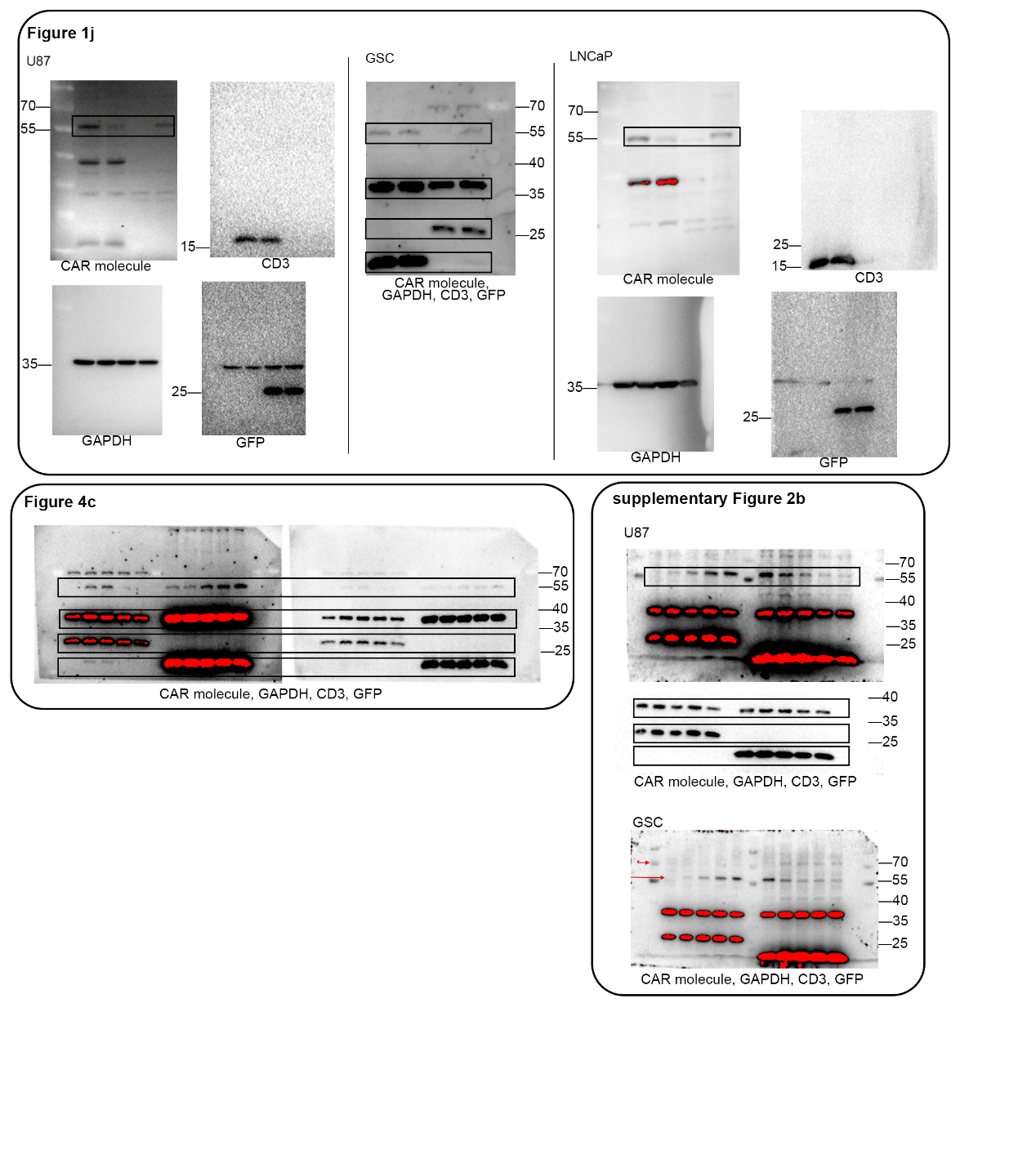

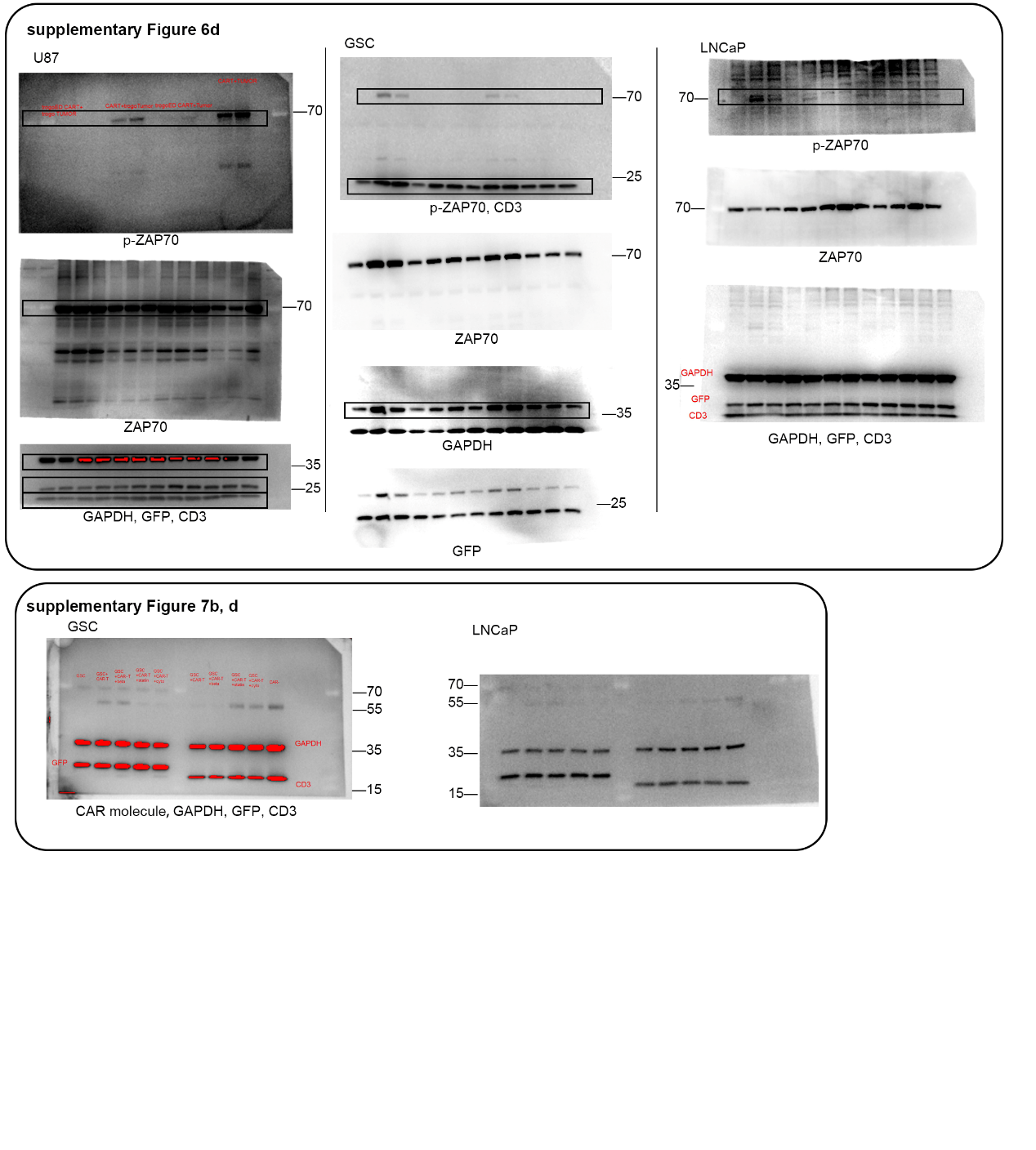
**

Supplement: Supplementary file 5 — All original films of Western blots [file 41392_2023_1708_MOESM5_ESM.docx]
